# Antiproliferative chromone derivatives induce K562 cell death through endogenous and exogenous pathways

**DOI:** 10.1080/14756366.2020.1740696

**Published:** 2020-03-18

**Authors:** Runwei Jiao, Fanxing Xu, Xiaofang Huang, Haonan Li, Weiwei Liu, Hao Cao, Linghe Zang, Zhanlin Li, Huiming Hua, Dahong Li

**Affiliations:** a Key Laboratory of Structure-Based Drug Design and Discovery, Ministry of Education, School of Traditional Chinese Materia Medica, Shenyang Pharmaceutical University, Shenyang, P. R. China; b Wuya College of Innovation, Shenyang Pharmaceutical University, Shenyang, P. R. China; c School of Life Science and Biopharmaceutics, Shenyang Pharmaceutical University, Shenyang, P. R. China

**Keywords:** Chromone, nitric oxide, antiproliferative, selectivity, apoptosis

## Abstract

A series of furoxan derivatives of chromone were prepared. The antiproliferative activities were tested against five cancer cell lines HepG2, MCF-7, HCT-116, B16, and K562, and two normal human cell lines L-02 and PBMCs. Among them, compound **15a** exhibited the most potent antiproliferative activity. It was also found **15a** produced more than 8 µM of NO at the peak time of 45 min by Griess assay. Generally, antiproliferative activity is positively related to NO release to some extent. Further in-depth studies on apoptosis-related mechanisms showed that **15a** caused S-phase cell cycle arrest in a concentration-dependent manner and induced apoptosis significantly through mitochondria-related pathways. Human apoptosis protein array assay also demonstrated **15a** increased the expression levels of pro-apoptotic Bax, Bad, HtrA2 and Trail R2/DR5. The expression of catalase and cell cycle blocker claspin were similarly up-regulated. In balance, **15a** induced K562 cells death through both endogenous and exogenous pathways.

## Graphical Abstract

**Figure:**
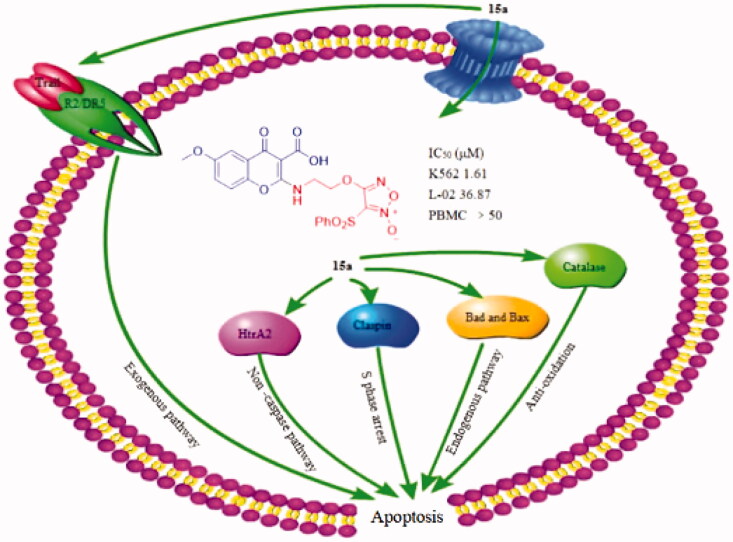

## 1. Introduction

From ancient times, various natural products have been used as traditional medicines and are rich sources of bioactive compounds^1^^,^^2^. Chromones (4*H*-chomen-4-one, 4*H*-1-benzopyran-4-one) are widely distributed oxygen-containing natural heterocyclic compounds from the plants of Polygonaceae, Umbelliferae, Sterculiaceae, Rhamnaceae, Liliaceae, Asteraceae etc., with a benzoylated *γ*-pyrone ring which is a part of the structure of flavonoid skeletons^3–5^. It is recognised as a privileged structure and a useful template for the design of novel compounds with potential pharmacological interest, particularly in the field of neurodegenerative^6^^,^^7^, inflammatory^8^^,^^9^, biocidal^10^, immune-stimulatory^11^, infectious diseases^12–14^, as well as diabetes^15^ and cancer^16–21^. With respect to antitumor activity, chromones demonstrate toxicity against many kinds of tumour cells, including cervical epithelioid carcinoma, breast adenocarcinoma, hepatoma carcinoma, lung cancer, leukaemia, colon cancer and so on. The antiproliferative mechanisms involve cytotoxicity, anti-metastasis, anti-angiogenesis, chemoprevention, immunomodulation and so forth^22–27^.

**Figure 1.**
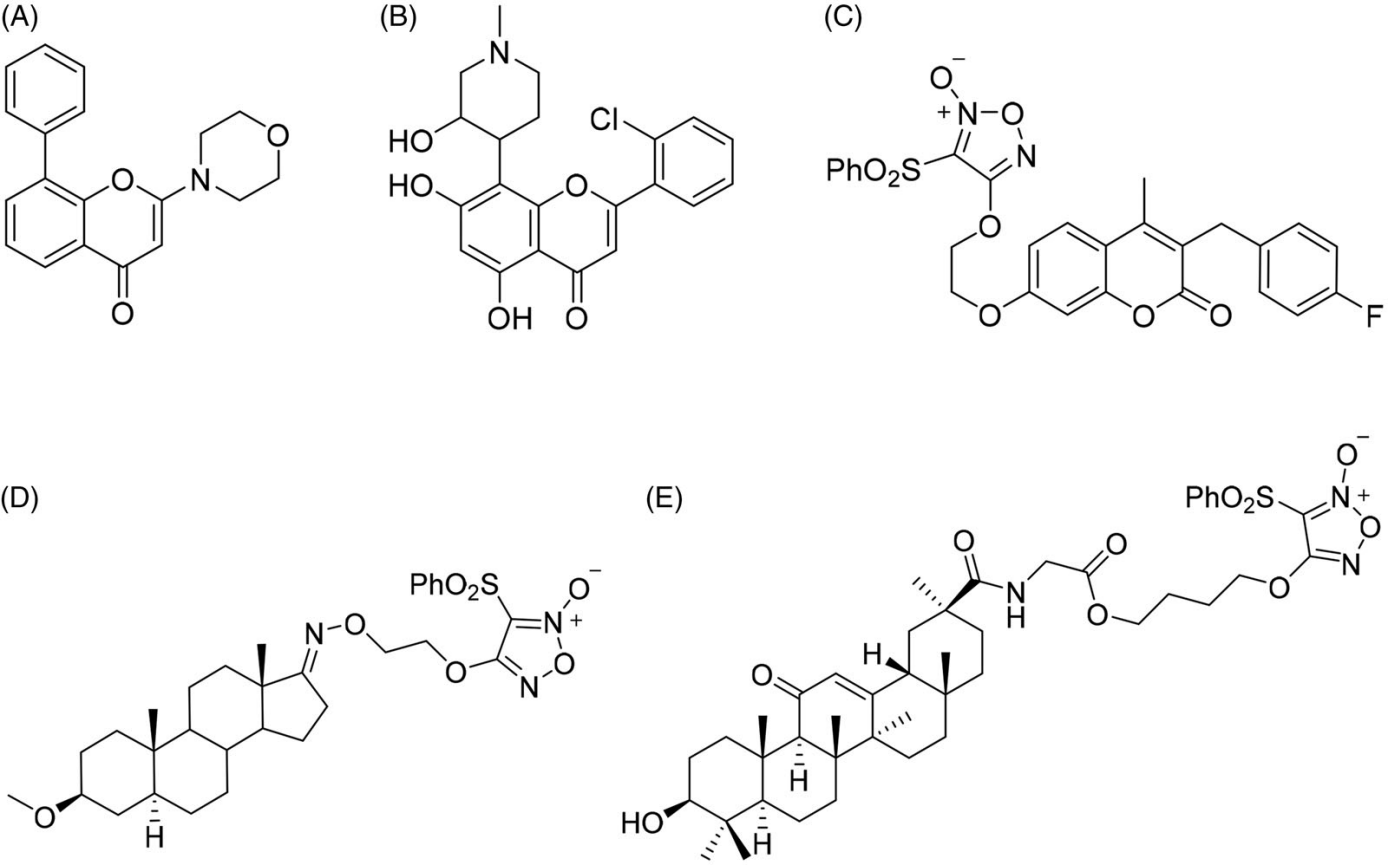
The chemical structures of reported chromone and furoxan derivatives. (A,B) Chromone derivatives; (C–E) furoxan-based NO donor derivatives.

Chromone has emerged as one of the most important synthetic scaffolds for their antitumor activity. Chromone derivative LY294002 (2-morpholino-8-phenyl-4*H*-chromen-4-one, Figure 1(A)) is a synthetic protein kinase inhibitor through the blockage of phosphatidylinositol-3-kinase (PI3K) signalling pathway^26^^,^^28^^,^^29^. Flavopiridol (2-(2-chlorophenyl)-5,7-dihydroxy-8-((3*R*,4*S*)-3-hydroxy-1-methylpiperidin-4-yl)-4*H*-chromen-4-one, Figure 1(B)) is identified as the first cyclin-dependent kinase (CDK) inhibitor which blocks cell cycle progression and induces apoptotic cell death. It has entered Phase II clinical trials^30^^,^^31^.

Nitric oxide (NO) acts as an important biological signalling molecule in a large variety of physiological processes, including neurotransmission, blood pressure regulation, defence mechanisms, smooth muscle relaxation and tumour growth inhibition^32–34^. Over the past decade, major advances of NO in cancer pathogenesis have been witnessed, suggesting an exciting future in the medical field^35^^,^^36^. Moreover, codelivery of NO with chemotherapeutic drugs enhances the suppression of tumour growth^37–40^, and it is also discovered to enhance the efficacy of other treatments, such as photodynamic therapy^41–43^, radiotherapy^44^^,^^45^ and ultrasound therapy^46^^,^^47^. However, NO has a half-life of only a few seconds in an aqueous environment, so NO release in specific targets is still a crucial challenge for antitumor therapy^48^^,^^49^. A wide range of NO donors have emerged as potential therapeutics to exploit the biological roles of NO^50–55^. Some NO donors produce high levels of NO with a wide range of halflives *in vitro* and *in vivo*, and have been widely used in drug research, especially the type of furoxan (Figure 1(C,D,E))^56–58^.

In this work, 16 chromone derivatives (**12a**–**d**, **13a**–**d**, **14a**–**d** and **15a**–**d)** with NO-releasing furoxan moiety were synthesised through different linkers. The linkers between the drug and NO donor influence NO releasing ability^56^. The antiproliferative activities against human tumour and normal cells were evaluated. Furthermore, in-depth apoptosis-related mechanisms of the most potent compound **15a**, including cell cycle progression, induction of apoptosis, changes of mitochondrial membrane potential and the expression of apoptosis-related proteins, were also explored.

## 2. Experimental

### 2.1. Chemistry

All starting materials and solvents were purchased from commercial suppliers and used without purification, unless otherwise noted. ^1^H and ^13^C NMR spectra were measured on Bruker AV400 spectrometers with tetramethylsilane (TMS) as the internal standard. Chemical shifts were reported in *δ* (ppm). High-resolution mass spectra were obtained on HClASS XEVOG2XSQTof in the ESI mode (HR-ESI-MS). All the spectra were in Supplemental data.

#### 2.1.1. General procedures for the synthesis of compounds 12a–d, 13a–d, 14a–d and 15a–d

A mixture of **10** or **11** (0.5 mmol) and HOBt (0.6 mmol) in anhydrous DMF (5 ml) was stirred at room temperature for 0.5 h. After the addition of 0.75 mmol 4-(2-aminoethoxy)-3-(phenylsulfonyl)-1,2,5-oxadiazole-2-oxide (**5a**), 4-(3-aminopropoxy)-3-(phenylsulfonyl)-1,2,5-oxadiazole-2-oxide (**5b**), 4-((1-aminopropan-2-yl)oxy)-3-(phenylsulfonyl)-1,2,5-oxadiazole-2-oxide (**5c**) or 3-(phenyl-sulfonyl)-4-(2-(piperazin-1-yl)ethoxy)-1,2,5-oxadiazole-2-oxide (**5d**) and EDCI (0.75 mmol) to the solution, the mixture was further stirred at room temperature for 3 h. Then, poured into 20 ml of H_2_O and extracted with EtOAc (3 × 20 ml). The organic layers were combined, washed with brine, dried over anhydrous Na_2_SO_4_ and concentrated *in vacuo*. The crude product was purified by silica gel column chromatography eluting with dichloromethane/methanol system.

#### 2.1.2 (2-(6-Methyl-4-oxo-4H-chromene-3-carboxamido)ethoxy)-3-(phenylsulfonyl)-1,2,5-oxadiazole 2-oxide (12a)

Yield: 52.6%. ^1^H NMR (400 MHz, DMSO-*d*_6_) *δ* (ppm): 9.45 (t, 1H, *J* = 6.1 Hz, –NH–), 9.05 (s, 1H, 3-ArH), 8.50 (s, 1H, 2′-ArH), 8.06–8.05 (d, 2H, *J* = 7.2 Hz, 3′,5′-ArH), 7.80 (s, 1H, 6′-ArH), 7.86–7.83 (t, 1H, *J* = 7.3 Hz, 4′-ArH), 7.77–7.74 (dd, 1H, *J* = 8.7, 1.9 Hz, 6-ArH), 7.72–7.68 (m, 2H, 5,8-ArH), 4.59–4.56 (t, 2H, *J* = 5.3 Hz, CH_2_–O), 3.83–3.79 (q, 2H, *J* = 11.0, 5.6 Hz, CH_2_–N–), 2.49 (s, 3H, –CH_3_); ^13^C NMR (100 MHz, DMSO-*d*_6_) *δ* (ppm): 176.7, 163.3, 163.1, 159.3, 154.4, 137.7, 136.9, 136.8, 136.6, 130.4, 128.9, 125.0, 123.8, 119.0, 115.5, 111.1, 70.5, 37.8, 21.0; HRMS (ESI) *m/z* calcd for C_21_H_16_N_3_O_8_S [M−H]^−^ 470.0658, found 470.0667.

#### 2.1.3 4-(2-((3-Carboxy-6-methyl-4-oxo-4H-chromen-2-yl)amino)ethoxy)-3-(phenylsulfonyl)-1,2,5-oxadiazole 2-oxide (12b)

Yield: 47.6%. ^1^H NMR (400 MHz, DMSO-*d*_6_) *δ* (ppm): 11.76 (s, 1H, –COOH), 8.62 (s, 1H, –NH–), 8.00–7.98 (d, 2H, *J* = 7.6 Hz, 2′,6′-ArH), 7.85–7.81 (t, 1H, *J* = 7.5 Hz, 4′-ArH), 7.77 (s, 1H, 5-ArH), 7.66–7.62 (t, 2H, *J* = 8.2 Hz, 3′,5′-ArH), 7.51–7.48 (dd, 1H, *J* = 8.4, 1.9 Hz, 6-ArH), 7.24–7.22 (s, 1H, 8-ArH), 4.69–4.67 (t, 2H, *J* = 4.6 Hz, CH_2_-O), 4.08–4.06 (t, 2H, *J* = 4.5 Hz, CH_2_–O), 2.38 (s, 3H, –CH_3_); ^13^C NMR (100 MHz, DMSO-*d*_6_) *δ* (ppm): 180.2, 164.1, 159.2, 153.0, 137.8, 136.6, 135.8, 133.8, 130.4, 128.8, 128.1, 126.0, 125.4, 117.3, 111.6, 96.8, 70.2, 49.2, 20.4; HRMS (ESI) *m/z* calcd for C_22_H_18_N_3_O_8_S [M−H]^−^ 484.0815, found 484.0803.

#### 2.1.4 4-(3-(6-Methyl-4-oxo-4H-chromene-3-carboxamido)propoxy)-3-(phenylsulfonyl)-1,2,5-oxadiazole 2-oxide (12c)

Yield: 48.8%. ^1^H NMR (400 MHz, DMSO-*d*_6_) *δ* (ppm): 9.27–9.24 (t, 1H, *J* = 5.8 Hz, –NH–), 9.03 (s, 1H, 3-ArH), 8.06–8.04 (d, 2H, *J* = 7.4 Hz, 2′,6′-ArH), 7.94 (s, 1H, 8-ArH), 7.91–7.87 (t, 1H, *J* = 7.5 Hz, 4′-ArH), 7.77–7.75 (m, 2H, 3′,5′-ArH), 7.73–7.71 (dd, 1H, *J* = 6.4, 1.7 Hz, 6-ArH), 7.69–7.67 (d, 1H, *J* = 8.5 Hz, 5-ArH), 4.49–4.46 (t, 2H, *J* = 6.0 Hz, CH_2_–O), 3.51–3.46 (q, 2H, *J* = 12.7, 6.48 Hz, –CH_2_–), 2.46 (s, 3H, –CH_3_), 2.08–2.03 (m, 2H, CH_2_–N); ^13^C NMR (100 MHz, DMSO-*d*_6_) *δ* (ppm): 176.7, 163.0, 162.8, 159.4, 154.4, 137.7, 136.7, 136.6, 130.5, 128.9, 125.1, 123.8, 118.9, 115.9, 111.0, 69.9, 35.7, 28.8, 21.0; HRMS (ESI) *m/z* calcd for C_22_H_18_N_3_O_8_S [M−H]^−^ 484.0815, found 484.0803.

#### 2.1.5 4-(3-((3-Carboxy-6-methyl-4-oxo-4H-chromen-2-yl)amino)propoxy)-3-(phenylsulfonyl)-1,2,5-oxadiazole 2-oxide (12d)

Yield: 37.5%. ^1^H NMR (400 MHz, DMSO-*d*_6_) *δ* (ppm): 11.62 (s, 1H, –COOH), 8.60–8.43 (m, 1H, –NH–), 8.06–8.04 (d, 2H, *J* = 7.9 Hz, 2′,6′-ArH), 7.94–7.88 (m, 1H, 4′-ArH), 7.77–7.69 (m, 3H, 3′,5′,5-ArH), 7.47–7.45 (m, 1H, 8-ArH), 7.21–7.17 (t, 1H, *J* = 9.6 Hz, 6-ArH), 4.50–4.46 (m, 2H, CH_2_–O), 3.74–3.69 (q, 2H, *J* = 12.7, 6.2 Hz, –CH_2_–), 2.36 (s, 3H, –CH_3_), 2.20–2.17 (m, 2H, CH_2_–N); ^13^C NMR (100 MHz, DMSO-*d*_6_) *δ* (ppm): 179.8, 163.3, 162.9, 161.4, 159.3, 152.8, 137.6, 136.6, 135.6, 130.5, 129.0, 125.3, 120.5, 117.2, 111.1, 96.4, 69.1, 47.6, 29.2, 20.8; HRMS (ESI) *m/z* calcd for C_25_H_23_N_4_O_8_S [M−H]^−^ 539.1237, found 539.1229.

#### 2.1.6 4-(2-(6-Methoxy-4-oxo-4H-chromene-3-carboxamido)ethoxy)-3-(phenylsulfonyl)-1,2,5-oxadiazole 2-oxide (13a)

Yield: 45.3%. ^1^H NMR (400 MHz, DMSO-*d*_6_) *δ* (ppm): 9.48–9.45 (t, 1H, *J* = 5.7 Hz –NH–), 9.05 (s, 1H, 3-ArH), 8.07–8.05 (d, 2H, *J* = 7.5 Hz, 2′,6′-ArH), 7.86–7.82 (t, 1H, *J* = 7.5 Hz, 4′-ArH), 7.79–7.76 (d, 1H, *J* = 8.9 Hz, 5-ArH), 7.71–7.67 (t, 2H, *J* = 8.0 Hz, 3′,5′-ArH), 7.55–7.53 (dd, 1H, *J* = 4.4, 2.9 Hz, 6-ArH), 7.51–7.50 (d, 1H, *J* = 3.1 Hz, 8-ArH), 4.59–4.56 (t, 2H, *J* = 5.1 Hz, CH_2_–O), 3.89 (s, 3H, –OCH_3_), 3.84–3.80 (q, 2H, *J* = 10.7, 5.4 Hz, CH_2_–N); ^13^C NMR (100 MHz, DMSO-*d*_6_) *δ* (ppm): 176.4, 163.1, 159.3, 157.8, 150.9, 137.7, 136.5, 130.4, 128.8, 124.9, 124.7, 120.9, 114.9, 111.1, 105.7, 70.6, 56.4, 37.8; HRMS (ESI) *m/z* calcd for C_21_H_16_N_3_O_9_S [M−H]^−^ 486.0607, found 486.0609.

#### 2.1.7 4-(2-((3-Carboxy-6-methoxy-4-oxo-4H-chromen-2-yl)amino)ethoxy)-3-(phenylsulfonyl)-1,2,5-oxadiazole 2-oxide (13b)

Yield: 35.7%. ^1^H NMR (400 MHz, DMSO-*d*_6_) *δ* (ppm): 11.74–11.71 (t, 1H, *J* = 6.6 Hz, –COOH), 8.73–8.57 (q, 1H, –NH–), 8.01–7.96 (m, 1H, 2′,6′-ArH), 7.85–7.80 (m, 1H, 4′-ArH), 7.66–7.61 (m, 2H, 3′,5′-ArH), 7.41–7.38 (m, 1H, 5-ArH), 7.30–7.28 (m, 2H, 6,8-ArH), 4.69–4.68 (m, 2H, CH_2_–O), 4.09–4.08 (m, 2H, CH_2_–N), 3.83 (s, 3H, –OCH_3_); ^13^C NMR (100 MHz, DMSO-*d*_6_) *δ* (ppm): 179.8, 177.5, 164.1, 159.1, 156.1, 155.9, 149.0, 137.7, 136.5, 130.4, 128.8, 122.3, 121.2, 118.8, 111.1, 107.6, 107.5, 70.1, 56.1, 40.6, 40.4, 40.2, 40.0, 39.8, 39.6, 39.4; HRMS (ESI) *m/z* calcd for C_22_H_18_N_3_O_9_S [M–H]^−^ 500.0764, found 500.0761.

#### 2.1.8 4-(3-(6-Methoxy-4-oxo-4H-chromene-3-carboxamido)propoxy)-3-(phenylsulfonyl)-1,2,5-oxadiazole 2-oxide (13c)

Yield: 33.9%. ^1^H NMR (400 MHz, DMSO-*d*_6_) *δ* (ppm): 9.28–9.25 (t, 1H, *J* = 5.7 Hz, –NH–), 9.03 (s, 1H, 3-ArH), 8.05–8.04 (d, 2H, *J* = 7.5 Hz, 2′,6′-ArH), 7.90–7.87 (t, 1H, *J* = 7.5 Hz, 4′-ArH), 7.77 (s, 1H, 5-ArH), 7.74–7.73 (m, 2H, 3′,5′-ArH), 7.51–7.49 (m, 2H, 6, 8-ArH), 4.50–4.47 (t, 2H, *J* = 6.0 Hz, CH_2_–O), 3.89 (s, 3H, –OCH_3_), 3.51–3.47 (m, 2H, CH_2_–N), 2.10–2.03 (m, 2H, –CH_2_–); ^13^C NMR (100 MHz, DMSO-*d*_6_) *δ* (ppm): 176.4, 162.8, 159.4, 157.7, 150.9, 137.6, 136.6, 130.5, 128.9, 124.9, 124.6, 120.8, 115.2, 111.0, 105.8, 69.9, 56.3, 35.8, 28.7; HRMS (ESI) *m/z* calcd for C_22_H_18_N_3_O_9_S [M−H]^−^ 500.0764, found 500.0782.

#### 2.1.9 4-(3-((3-Carboxy-6-methoxy-4-oxo-4H-chromen-2-yl)amino)propoxy)-3-(phenylsulfonyl)-1,2,5-oxadiazole 2-oxide (13d)

Yield: 56.6%. ^1^H NMR (400 MHz, DMSO-*d*_6_) *δ* (ppm): 11.61 (s, 1H, –COOH), 8.50 (s, 1H, –NH–), 8.06–8.04 (d, 2H, *J* = 7.5 Hz, 2′,6′-ArH), 7.92–7.89 (t, 1H, *J* = 7.4 Hz, 4′-ArH), 7.78–7.74 (t, 2H, *J* = 7.8 Hz, 3′,5′-ArH), 7.35 (s, 1H, 5-ArH), 7.21 (s, 2H, 6,8-ArH), 4.48–4.45 (t, 2H, *J* = 5.7 Hz, CH_2_–O), 3.80 (s, 3H, –OCH_3_), 3.17–3.16 (d, 2H, *J* = 2.8 Hz, CH_2_–N), 2.18–2.15 (m, 2H, –CH_2_–); ^13^C NMR (100 MHz, DMSO-*d*_6_) *δ* (ppm): 159.3, 155.9, 148.9, 137.6, 136.6, 130.5, 129.0, 126.0, 118.8, 111.1, 107.6, 96.4, 69.2, 56.1, 47.8, 29.2; HRMS (ESI) *m/z* calcd for C_25_H_23_N_4_O_9_S [M−H]^−^ 555.1186, found 555.1173.

#### 2.1.10 2-((1-(6-Methyl-4-oxo-4H-chromene-3-carboxamido)propan-2-yl)oxy)-3-(phenylsulfonyl)-1,2,5-oxadiazole 2-oxide (14a)

Yield: 28.1%. ^1^H NMR (400 MHz, DMSO-*d*_6_) *δ* (ppm): 9.43–9.40 (t, 1H, *J* = 5.5 Hz, –NH–), 9.04–9.03 (d, 1H, *J* = 6.5 Hz, 3-ArH), 8.07–8.05 (d, 2H, *J* = 7.4 Hz, 2′,6′-ArH), 7.97–7.96 (d, 1H, *J* = 5.6 Hz, 8-ArH), 7.86–7.82 (t, 1H, *J* = 7.4 Hz, 4′-ArH), 7.76–7.74 (dd, 1H, *J* = 8.7 Hz, 6-ArH), 7.72–7.67 (m, 3H, 3′,5′, 5-ArH), 5.18–5.14 (m, 1H, –CH–), 3.86–3.80, 3.65–3.59 (m, 2H, –CH_2_–N–), 2.48 (s, 3H, ArH-CH_3_), 1.40–1.39 (d, 3H, *J* = 6.3 Hz, –CH–CH_3_); ^13^C NMR (100 MHz, DMSO-*d*_6_) *δ* (ppm): 176.8, 163.3, 163.2, 158.8, 154.4, 137.6, 136.9, 136.5, 130.4, 128.9, 126.0, 125.0, 123.7, 119.0, 115.5, 78.5, 42.6, 21.0, 17.2; HRMS (ESI) *m/z* calcd for C_21_H_16_N_3_O_9_S [M–H]^−^ 486.0607, found 486.0609.

#### 2.1.11 4-((1-((3-Carboxy-6-methyl-4-oxo-4H-chromen-2-yl)amino)propan-2-yl)oxy)-3-(phenylsulfonyl)-1,2,5-oxadiazole 2-oxide (14b)

Yield: 35.6%. ^1^H NMR (400 MHz, DMSO-*d*_6_) *δ* (ppm): 11.72 (s, 1H, –COOH), 8.58 (s, 1H, –NH–), 8.00–7.99 (d, 2H, *J* = 7.2 Hz, 2′,6′-ArH), 7.84–7.80 (t, 1H, *J* = 7.2 Hz, 4′-ArH), 7.74 (s, 1H, 5-ArH), 7.65–7.61 (t, 2H, *J* = 7.4 Hz, 3′,5′-ArH), 7.48–7.47 (d, 1H, *J* = 7.3 Hz, 8-ArH), 7.21–7.19 (d, 1H, *J* = 8.9 Hz, 6-ArH), 5.26–5.24 (m, 1H, CH–O), 4.11–4.00 (m, 3H, –CH_2_–), 2.37 (s, 3H, Ar–CH_3_), 1.40–1.39 (d, 3H, CH-CH_3_); ^13^C NMR (100 MHz, DMSO-*d*_6_) *δ* (ppm): 180.6, 164.2, 163.7, 160.1, 153.6, 138.4, 137.4, 136.4, 134.4, 131.3, 129.8, 126.1, 121.3, 118.0, 111.9, 97.2, 69.9, 48.4, 30.0, 21.6; HRMS (ESI) *m/z* calcd for C_22_H_18_N_3_O_9_S [M–H]^−^ 500.0764, found 500.0783.

#### 2.1.12 4-(2-(4-(6-Methyl-4-oxo-4H-chromene-3-carbonyl)piperazin-1-yl)ethoxy)-3-(phenylsulfonyl)-1,2,5-oxadiazole 2-oxide (14c)

Yield: 29.7%. ^1^H NMR (400 MHz, DMSO-*d*_6_) *δ* (ppm): 8.51 (s, 1H, 3-ArH), 8.03–8.01 (d, 2H, *J* = 7.4 Hz, 2′,6′-ArH), 7.90–7.86 (t, 2H, *J* = 7.4 Hz, 4′,6-ArH), 7.76–7.72 (t, 2H, *J* = 8.0 Hz, 3′,5′-ArH), 7.69–7.66 (dd, 1H, *J* = 8.6, 1.9 Hz, 5-ArH), 7.62–7.60 (d, 1H, *J* = 8.6 Hz, 8-ArH), 4.54–4.51 (t, 2H, *J* = 5.0 Hz, –CH_2_–O), 3.58–3.56 (t, 2H, *J* = 4.9 Hz, CON–CH_2_), 3.27–3.24 (t, 2H, *J* = 5.4 Hz, CON–CH_2_), 2.81–2.79 (t, 2H, *J* = 5.1 Hz, –N–CH_2_–), 2.55–2.53 (t, 2H, *J* = 5.2 Hz, –CH_2_–N–C), 2.48–2.45 (t, 2H, *J* = 5.0 Hz, –CH_2_–N–C), 2.44 (s, 3H, –CH_3_); ^13^C NMR (100 MHz, DMSO-*d*_6_) *δ* (ppm): 173.8, 162.5, 159.4, 156.5, 154.5, 137,8, 136.6, 136.2, 136.1, 130.5, 128.7, 127.8, 125.0, 123.8, 122.6, 118.9, 69.7, 55.9, 53.4, 52.8, 47.1, 41.9, 20.9; HRMS (ESI) *m/z* calcd for C_22_H_18_N_3_O_9_S [M–H]^−^ 500.0764, found 500.0776.

#### 2.1.13 4-(2-(4-(3-Carboxy-6-methyl-4-oxo-4H-chromen-2-yl)piperazin-1-yl)ethoxy)-3-(phenlsulfonyl)-1,2,5-oxadiazole 2-oxide (14d)

Yield: 27.6%. ^1^H NMR (400 MHz, DMSO-*d*_6_) *δ* (ppm): 14.1 (s, 1H, –COOH), 8.03 (m, 2H, 2′,6′-ArH), 7.92–7.89 (m, 1H, 4′-ArH), 7.86–7.83 (d, 1H, *J* = 9.0 Hz, 5-ArH), 7.79–7.75 (m, 3H, 3′,5′,8-ArH), 7.19–7.17 (dd, 1H, *J* = 8.3 Hz, 6-ArH), 4.56–4.54 (t, 1H, *J* = 5.1 Hz, CH–O), 3.54–3.52 (t, 3H, *J* = 4.6 Hz, CH–O, Ar–N–CH_2_), 3.17 (s, 2H, N–CH_2_), 2.86–2.84 (t, 2H, *J* = 5.2 Hz, N-CH_2_), 2.62–2.60 (t, 4H, *J* = 4.1 Hz, –CH_2_–N–CH_2_–), 2.25 (s, 3H, –CH_3_); ^13^C NMR (100 MHz, DMSO-*d*_6_) *δ* (ppm): 191.2, 160.7, 154.3, 137.8, 136.6, 135.1, 130.6, 129.2, 128.7, 128.1, 127.8, 126.0, 120.2, 117.7, 111.0, 89.8, 70.3, 69.9, 55.7, 53.5, 49.1, 29.5, 20.6; HRMS (ESI) *m/z* calcd for C_25_H_23_N_4_O_9_S [M–H]^−^ 555.1186, found 555.1171.

#### 2.1.14 4-((1-(6-Methoxy-4-oxo-4H-chromene-3-carboxamido)propan-2-yl)oxy)-3-(phenylsulfonyl)-1,2,5-oxadiazole 2-oxide (15a)

Yield: 29.5%. ^1^H NMR (400 MHz, DMSO-*d*_6_) *δ* (ppm): 9.45–9.42 (t, 1H, *J* = 11.6 Hz, –NH–), 9.04 (s, 1H, 3-ArH), 8.07–8.06 (d, 2H, *J* = 7.4 Hz, 2′,6′-ArH), 7.86–7.82 (t, 1H, *J* = 7.5 Hz, 4′-ArH), 7.79–7.77 (m, 1H, 5-ArH), 7.71–7.67 (t, 2H, *J* = 8.0 Hz, 3′,5′-ArH), 7.54–7.52 (q, 2H, *J* = 7.8, 3.2 Hz, 6,8-ArH), 5.18–5.14 (m, 1H, CH–O), 4.54–4.52 (m, 1H, –CH–N–), 3.90 (s, 3H, Ar–CH_3_), 3.66–3.59 (m, 1H, –CH–N–), 1.41–1.40 (d, 3H, CH–CH_3_); ^13^C NMR (100 MHz, DMSO-*d*_6_) *δ* (ppm): 176.5, 163.2, 163.1, 158.8, 157.8, 150.9, 137.6, 136.5, 130.4, 128.9, 124.8, 120.9, 114.9, 111.1, 105.6, 78.5, 56.4, 42.6, 17.2; HRMS (ESI) *m/z* calcd for C_21_H_16_N_3_O_10_S [M–H]^−^ 502.0556, found 502.0544.

#### 2.1.15 4-((1-((3-Carboxy-6-methoxy-4-oxo-4H-chromen-2-yl)amino)propan-2-yl)oxy)-3-(phenylsulfonyl)-1,2,5-oxadiazole 2-oxide (15b)

Yield: 22.1%. ^1^H NMR (400 MHz, DMSO-*d*_6_) *δ* (ppm): 11.7 (s, 1H, –COOH), 8.65 (s, 1H, –NH–), 8.00 (m, 2H, 2′,6′-ArH), 7.84–7.81 (t, 1H, *J* = 7.2 Hz, 4′-ArH), 7.65–7.63 (t, 2H, *J* = 7.5 Hz, 3′,5′-ArH), 7.37 (s, 1H, 5-ArH), 7.28 (s, 2H, 6,8-ArH), 5.28–5.21 (m, 1H, CH–O), 4.06–3.82 (m, 2H, –CH_2_–), 3.82 (s, 3H, Ar-CH_3_), 1.41–1.39 (d, 3H, CH–CH_3_); ^13^C NMR (100 MHz, DMSO-*d*_6_) *δ* (ppm): 179.9 164.3, 163.2, 158.6, 155.9, 149.0, 137.6, 136.6, 130.3, 128.9, 128.1, 125.9, 122.5, 121.1, 118.9, 111.1, 107.4, 78.0, 70.2, 56.1, 29.5, 16.6; HRMS (ESI) *m/z* calcd for C_22_H_18_N_3_O_10_S [M–H]^−^ 516.0713, found 516.0728.

#### 2.1.16 4-(2-(4-(6-Methoxy-4-oxo-4H-chromene-3-carbonyl)piperazin-1-yl)ethoxy)-3-(phenylsulfonyl)-1,2,5-oxadiazole 2-oxide (15c)

Yield: 29.9%. ^1^H NMR (400 MHz, DMSO-*d*_6_) *δ* (ppm): 8.52 (s, 1H, 3-ArH), 8.04–8.01 (m, 2H, 2′,6′-ArH), 7.90–7.87 (t, 1H, *J* = 7.5 Hz, 4′-ArH), 7.77–7.73 (t, 2H, *J* = 8.1 Hz, 3′,5′-ArH), 7.69–7.67 (dd, 1H, *J* = 7.8, 1.9 Hz, 6-ArH), 7.46–7.43 (m, 2H, 5, 8-ArH), 4.55–4.52 (t, 2H, *J* = 10.1 Hz, CH_2_–O), 3.87 (s, 3H, –OCH_3_), 3.60–3.57 (t, 2H, *J* = 4.4 Hz, –CON–CH_2_), 3.28–3.26 (t, 2H, *J* = 7.5 Hz, –CON–CH_2_), 2.82–2.80 (t, 2H, *J* = 5.0 Hz, –N–CH_2_), 2.56–2.54 (t, 2H, *J* = 5.0 Hz, –CH_2_–N–C), 2.49–2.47 (t, 2H, *J* = 4.9 Hz, –CH_2_–N–C); ^13^C NMR (100 MHz, DMSO-*d*_6_) *δ* (ppm): 173.5, 162.6, 159.4, 157.3, 156.4, 151.0, 137.8, 136.6, 130.5, 128.7, 124.9, 124.2, 121.9, 120.7, 110.9, 105.4, 69.7, 56.3, 55.9, 53.4, 52.8, 47.1, 41.9; HRMS (ESI) *m/z* calcd for C_22_H_18_N_3_O_10_S [M–H]^−^ 516.0713, found 516.0714.

#### 2.1.17 4-(2-(4-(3-Carboxy-6-methoxy-4-oxo-4H-chromen-2-yl)piperazin-1-yl)ethoxy)-3-(phenylsulfonyl)-1,2,5-oxadiazole 2-oxide (15d)

Yield: 37.8%. ^1^H NMR (400 MHz, DMSO-*d*_6_) *δ* (ppm): 13.9 (s, 1H, -COOH), 8.04 (m, 2H, 2′,6′-ArH), 7.92–7.87 (m, 2H, 4′,5-ArH), 7.79–7.75 (m, 3H, 3′,5′,8-ArH), 7.03–7.00 (dd, 1H, *J* = 8.9, 3.0 Hz, 6-ArH), 4.56–4.54 (t, 2H, *J* = 5.0 Hz, CH_2_–O), 3.75 (s, 3H, –OCH_3_), 3.55 (s, 4H, Ar–N–(CH_2_)_2_), 2.86–2.84 (t, 2H, *J* = 5.0 Hz, –N–CH_2_), 2.62–2.60 (t, 4H, *J* = 4.6 Hz, –N–(CH_2_)_2_); ^13^C NMR (100 MHz, DMSO-*d*_6_) *δ* (ppm): 190.8, 157.2, 156.9, 154.6, 151.5, 137.8, 136.6, 130.6, 128.7, 126.0, 121.6, 120.6, 120.4, 118.6, 113.0, 105.2, 89.8, 69.9, 60.4, 56.4, 56.2, 55.7, 29.5; HRMS (ESI) *m/z* calcd for C_25_H_23_N_4_O_10_S [M–H]^−^ 571.1135, found 571.1122.

### 2.2. MTT assay

The colorimetric MTT (3-(4,5-dimethylthiazol-2-yl)-2,5-diphenyltetrazolium bromide) assay was used to measure the cell viability of all of the above cell lines following treatment with target hybrids. Exponentially growing cells were added into 96-well plates at a concentration of 2000–4000 cells per well. Following attachment the cells were treated with varying concentrations (64, 16, 4, 1, 0.25, 0.0625 and 0.015625 µM) of target compounds in media and were kept for incubation for 72 h. MTT (20 µL, 5 mg/mL in PBS) was added to each well and the cells were incubated for another 3 h at 37 °C. Then the medium was removed, followed by the addition of 150 µL DMSO for each well. After that, the absorbance (OD) data of each well at 570 nm wavelength were measured by a Microplate Reader (BIO-RAD), and half inhibition rates (IC_50_) were calculated^59^^,^^60^.

### 2.3. No releasing test

The levels of NO produced by each compound were determined by colorimetric assay using a nitrite colorimetric assay kit (Beyotime, China) according to the manufacturer’s instructions. Incubated in phosphate buffer (pH 7.4) containing 2% dimethyl sulfoxide and 10^−5 ^M test compound (1 ml of 0.2 mM solution in 0.1 M phosphate buffer, pH 7.4) with freshly prepared L-cysteine (1 ml of 3.6 mM solution in 0.1 M phosphate buffer, pH 7.4) at 37 °C for 1, 2 and 3 h without air. After exposure to air for 10 min at 25 °C, aliquots of Griess reagent I (50 µL) and Griess reagent II (50 µL) were added to an equal volume (50 µL) of the incubation solution of each test compound. After 10 min, the absorbance was measured at 540 nm. The nitrite absorbance versus concentration curve was prepared using 1 M sodium nitrite solution under the same experimental conditions. The concentration of NO formed by a single test compound was calculated using different concentrations of nitrite as a standard.

### 2.4. Stability of 15a

Compound **15a** was dissolved in culture medium to a final concentration of 100 µM from 50 mM stock solution in DMSO. The solutions were incubated at 37 °C. An aliquot (20 µL) of the incubation mixture was taken at different time points (0, 1, 2, 3, 4, 5, 6, 8, and 12 h). And the components were analysed by HPLC equipped with a C18 reverse phase column (Shimadzu, LC-6 AD) with the flow rate of 0.5 ml/min methanol-water (60: 40–100: 0) and detection at UV 230 nm.

### 2.5. Cell cycle study

The cells in the logarithmic growth phase were digested into six-well plates. The next day, cells were treated with different concentrations (0, 0.8, 1.6, 3.2 µM) of compound **15a**. After 72 h, 5 × 105 cells were collected and fixed with a volume fraction of 70% ethanol for 2 h (or overnight), washed the fixative with PBS, added 100 µL RNase A in 37 °C water bath for 30 min, and finally 400 µL of PI stain was added, after 30 min under the dark at 4 °C, the cytometry was used to record the red fluorescence at the excitation wavelength of 488 nm, and the cell cycle distribution of the DNA content was evaluated.

### 2.6. Hoechst 333258 staining

K562 cells were incubated with different concentrations (0, 0.8, 1.6 and 3.2 µM) of **15a** for a period of time, the medium containing the compound was removed, the cell smear was naturally dried, and immersed in 4% paraformaldehyde for 30 min or overnight to improve cell permeability, then soaked in PBS, washed for three times, added appropriate amount of Hoechst 33258 staining solution, fully covered, allowed to stand at room temperature for 10 min, finally immersed in PBS again, washed three times and sealed. Anti-fluorescence quenching after the liquid was sealed, the staining results were observed under a fluorescence microscope.

### 2.7. Analysis of cellular apoptosis

The cells were placed in six-well plates and incubated at 37 °C for 24 h, after which K562 cells were treated with different concentrations (0, 0.8, 1.6, 3.2 µM) of **15a** for 24 h, washed with PBS, centrifuged, and collected. 500 µL of binding buffer suspension cells, 5 µL of Annexin V-FITC, and 5 µL of PI were mixed incubated at room temperature, protected from light, and reacted for 5–15 min, then analysed with flow cytometry instrument to detect cell apoptosis.

### 2.8. Cell mitochondrial membrane potential assay

K562 cells were cultured for 48 h in six-well plates with different concentrations of **15a** (0, 0.8, 1.6 and 3.2 µM), then washed with PBS and stained with JC-1 in the dark at room temperature. Flow cytometry was used to measure the number of cells with collapsed mitochondrial membrane potential.

### 2.9. Quantitation of cellular proteins involved in apoptosis

The relative expression levels of 35 apoptosis-related proteins were evaluated using Human Apoptosis Array kit (R&D Systems, Abingdon, UK) in K562 cells. Proteins were extracted according to the manufacturer’s protocol from cells treated for 24 h with compound **15a** (2 µM). The tool is fast, sensitive and economical, with 2.0 ml of array buffer added to each well, followed by array, The capture antibodies are retained in their specific locations, then incubated, transferred, diluted, added to Streptavidin-HRP, shaken. The platform shaker was incubated on the plate for 30 min, then the membrane was removed, 1 ml of the prepared Chemi Reagent Mix was evenly pipetted onto each membrane, incubated, the excess Chemi Reagent Mix was removed, and the membrane was placed in a self-developing film cartridge, exposed to X-ray film for 1–10 min. The positive signals seen on developed film can be quickly identified by placing the transparency overlay template on the array image and aligning it with the pairs of reference spots in three corners of each array. Creating templates, exporting files, averaging signals, finding backgrounds, comparing corresponding signals on the array to determine relative changes in apoptosis-related protein levels.

## 3. Results and discussion

### 3.1. Chemistry

**Scheme 1.**
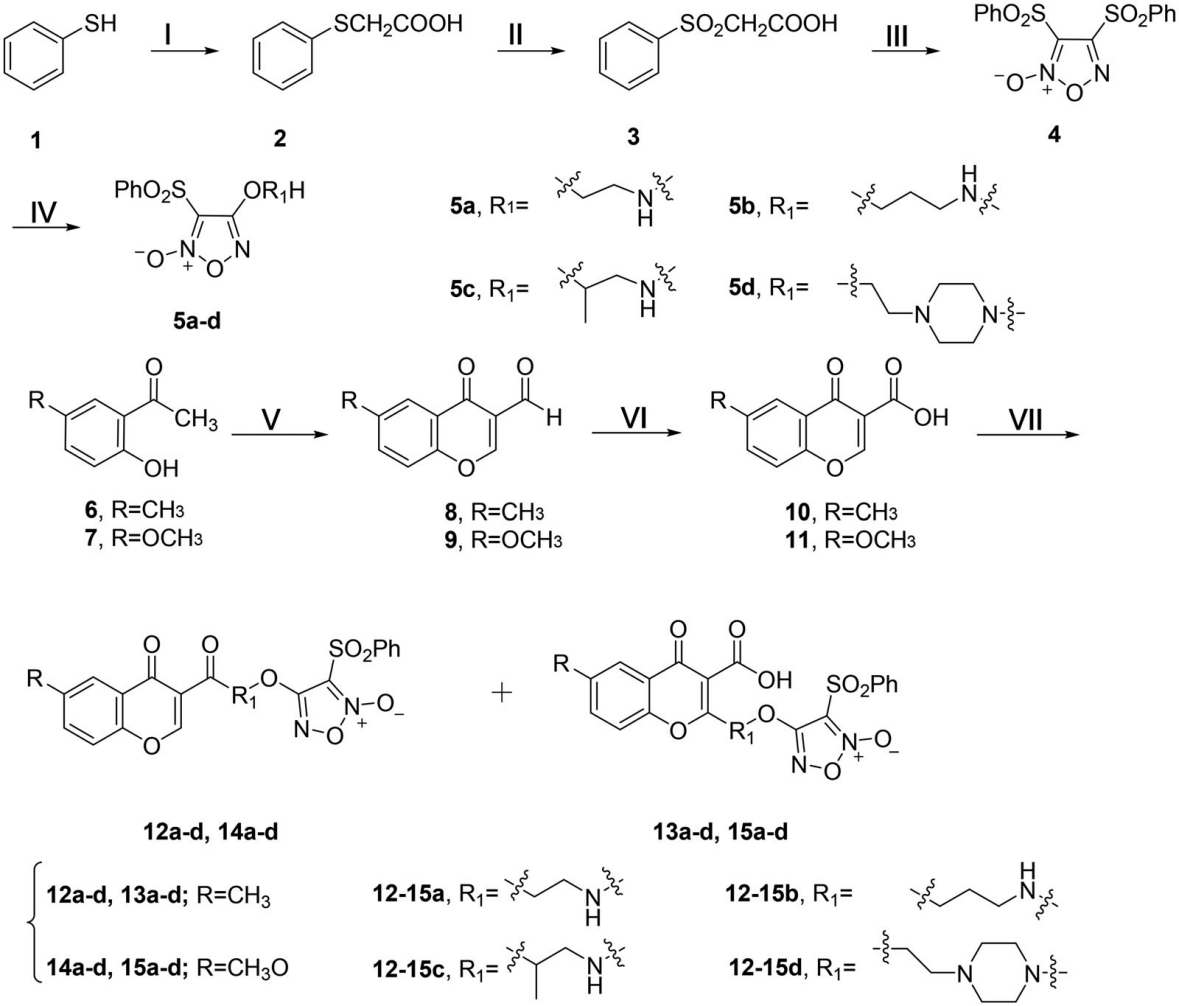
Synthesis of **5a–d, 12a–d, 13a–d, 14a–b** and **15a–b**. Reagents and conditions: (**I**) ClCH_2_COOH, NaOH (aq), reflux, 2 h; (**II**) 30% H_2_O_2_, AcOH, rt, 3 h; (**III**) fuming HNO_3_, 90 °C, 4 h; (**IV**) aminoalcohol, THF, NaH, 0 °C, 4 h; (**V**) POCl_3_, DMF, −10 °C, 15 h; (**VI**) H_3_NSO_3_, NaClO_2_, 0 °C, 12 h; (**VII**) **5a–d**, HOBt, EDCI, DMF, rt, 3 h.

The synthetic routine of target compounds is illustrated in Scheme 1. Compound **5** was synthesised in a three-step sequence according to the literature^61^, and then converted to various monophenylsulfonylfuroxans (**5a**–**d**) by the treatment with corresponding amino-substituted alcohol, ethanolamine, 3-aminopropanol, 1-aminopropan-2-ol and *N*-(2-hydroxyethyl)piperazine (Scheme 1).

Compounds **8** and **9** were synthesised from 2-hydroxy-5-methoxyacetophenone (**6**) and 2-hydroxy-5-methylacetophenone (**7**), respectively, by using the Vilsmeier Haack reagent (POCl_3_ and DMF) at −10 °C. Then the aldehyde group was oxidised to carboxylic acid by sulphamic acid and sodium chlorite^62^. Finally, the furoxan intermediates (**5a**–**d**) were reacted with the derivatives (**10** and **11**) of chromone to give the target compounds **12a**–**d** plus **13a**–**d** and **14a**–**d** plus **15a**–**d**, respectively (Scheme 1). The structures of all the derivatives were confirmed by ^1^H NMR, ^13^C NMR and high resolution mass spectrum (HR-MS).

### 3.2. Biological evaluation

#### 3.2.1. Antiproliferative activity

Target chromone/furoxan hybrids (**12a**-**d**, **13a**-**d**, **14a**-**d** and **15a**-**d**) were evaluated for their inhibitory effects against five different human cancer cell lines (hepatoma HepG2, breast carcinoma MCF-7, colorectal carcinoma HCT-116, melanoma B16 and chronic myeloid leukaemia K562), with the reference fluorouracil (5-Fu)^63–66^. Meanwhile, the activities against the human normal hepatic L-02 cell line and peripheral blood mononuclear cells (PBMCs) were also evaluated since a potential anticancer drug candidate would be better to show selective cytotoxicity between malignant and normal cells.

As shown in Table 1, most target compounds displayed more potent inhibitory activities than corresponding chromone parent compound. For instance, the antiproliferative activities of the furoxan hybrids **12d**, **14c**, **14d** and **15a** showed IC_50_ values ranging from 1.61 to 15.92 µM against five cancer cell lines. Especially, they were more sensitive to K562 (IC_50_ 1.61–4.94 µM) and HepG2 (IC_50_ 4.86–7.91 µM) cells. Among **12a**, **13a**, **14a** and **15a**, **14a** and **15a** with a methoxy group at the 5-position of chromone displayed more potent antiproliferative activities against HepG2, HCT-116 and K562 cells than corresponding ones with methyl group. The results were in accord with previous literatures that bulky methoxy group was preferred for antiproliferative activity^67^^,^^68^. In addition, the presence of carboxyl groups also enhanced antiproliferaitve activity, which were more easily salified to increase water solubility. Furthermore, **12d** and **14d** with a nitrogen-containing heterocyclic ring showed stronger antiproliferative activities. Other compounds generally followed the above rules. The target derivatives were sensitive to K562 cell line, of which **15a** displayed the most potent antiproliferative activity with an IC_50_ value of 1.61 µM.

**Table 1.** The antiproliferative effects of the target compounds against different human cancer and normal cell lines.

Compound	IC_50_ (μM)^a^						
	HepG2	MCF-7	HCT-116	B16	K562	L-02	PBMC
**10**	>50	>50	>50	>50	>50	>50	>50
**11**	>50	>50	>50	>50	>50	>50	>50
**5a**	21.76 ± 1.35	>50	29.86 ± 1.47	27.94 ± 1.23	18.86 ± 0.73	>50	>50
**5b**	17.82 ± 1.43	>50	18.93 ± 1.67	22.85 ± 1.46	20.34 ± 1.88	>50	>50
**5c**	>50	>50	>50	>50	>50	>50	>50
**5d**	>50	>50	>50	>50	>50	>50	>50
**12a**	>50	>50	>50	>50	>50	>50	>50
**13a**	10.59 ± 0.72	22.14 ± 1.38	13.19 ± 0.53	16.84 ± 0.74	5.89 ± 0.26	>50	>50
**14a**	19.89 ± 1.31	>50	29.57 ± 1.48	27.94 ± 1.23	13.56 ± 0.85	>50	>50
**15a**	4.86 ± 0.39	13.71 ± 0.33	6.74 ± 0.90	9.72 ± 0.52	1.61 ± 0.18	36.87 ± 0.62	>50
**12b**	21.34 ± 1.58	26.59 ± 1.60	26.87 ± 1.61	31.44 ± 1.34	12.46 ± 1.03	>50	>50
**13b**	22.56 ± 1.39	24.83 ± 1.28	26.92 ± 1.40	26.82 ± 1.22	13.55 ± 0.76	>50	>50
**14b**	>50	>50	>50	>50	>50	>50	>50
**15b**	>50	>50	>50	>50	>50	>50	>50
**12c**	21.47 ± 1.23	>50	25.36 ± 1.76	26.87 ± 1.08	12.46 ± 0.77	>50	>50
**13c**	>50	>50	>50	>50	>50	>50	>50
**14c**	5.67 ± 0.42	12.84 ± 1.46	7.93 ± 0.44	10.56 ± 0.53	2.98 ± 0.14	41.27 ± 2.58	>50
**15c**	25.64 ± 1.57	37.59 ± 2.24	22.39 ± 1.72	23.54 ± 1.61	8.93 ± 0.36	>50	>50
**12d**	7.91 ± 0.40	15.92 ± 0.66	8.64 ± 0.58	9.96 ± 0.67	4.94 ± 0.25	33.64 ± 2.95	>50
**13d**	>50	>50	>50	>50	>50	>50	>50
**14d**	5.38 ± 0.19	13.85 ± 0.49	7.87 ± 0.32	11.73 ± 0.52	2.75 ± 0.17	28.90 ± 1.43	>50
**15d**	9.56 ± 2.37	16.89 ± 1.29	12.86 ± 1.02	35.02 ± 1.25	4.07 ± 0.39	>50	>50
5-Fu	32.57 ± 1.98	26.65 ± 1.92	6.86 ± 0.37	12.62 ± 1.06	3.94 ± 0.17	>50	>50

^a^ IC_50_: Half inhibitory concentrations measured by the MTT assay. The values are expressed as average ± standard deviation of three independent experiments.

In addition, all the target compounds exhibited weak antiproliferative activities with IC_50_ values greater than 50 µM against PBMCs and above 28.90 µM against L-02 cells, which showed good selectivity between tumour and normal cells.

#### 3.2.2. No releasing ability in vitro

**Figure 2.**
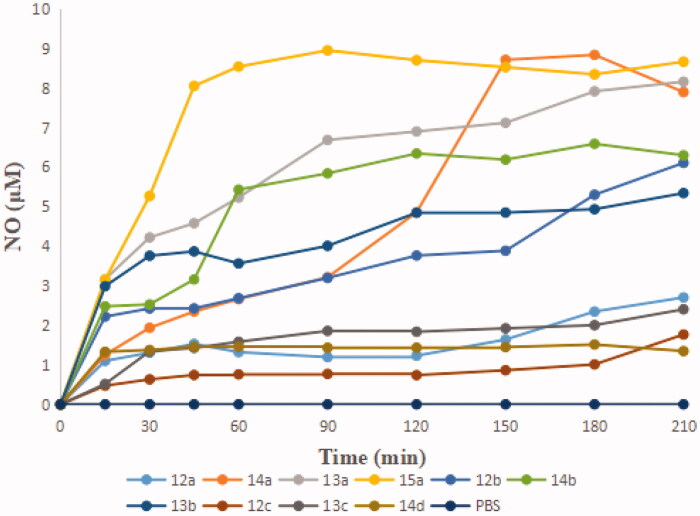
The amounts of NO released by the target compounds **12a–d**, **13a–d**, **14a–d,** and **15a–d**.

The levels of NO released were tested by Griess assay. As shown in Figure 2, the amounts were basically consistent with the potency of antiproliferative activity, such as **13a**-**b**, **14b** and **15a**. In addition, the faster the amounts of NO released reached the peak, the stronger activity exhibited. **15a**, which showed the strongest growth inhibitory activity, produced more than 8 µM of NO at the peak time of 45 min. Generally, the antiproliferative activity is somehow robust related to the amount of NO released.

#### 3.2.3. Stability of 15a

**Figure 3.**
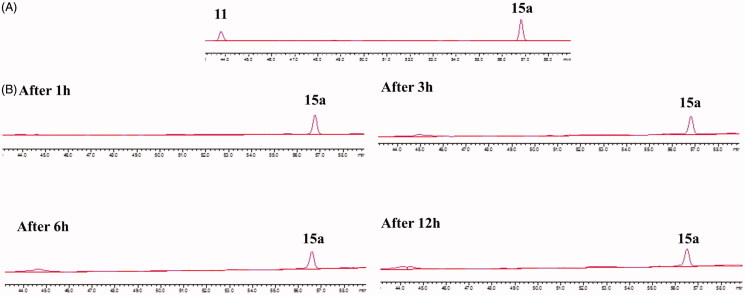
HPLC of (A) **11** and **15a** in MeOH; (B) **15a** in cell-free culture medium (cRPMI-1640) after incubation for 1, 3, 6, and 12 h.

To test that if **15a** was easy to hydrolyse, its stability was studied in cRPMI-1640 culture media supplemented with FBS under cell-free conditions. The results of HPLC analysis were summarised in Figure 3. It was observed that **15a** was relatively stable in cRPMI-1640 culture media within 12 h.

#### 3.2.4. Cell cycle analysis

**Figure 4.**
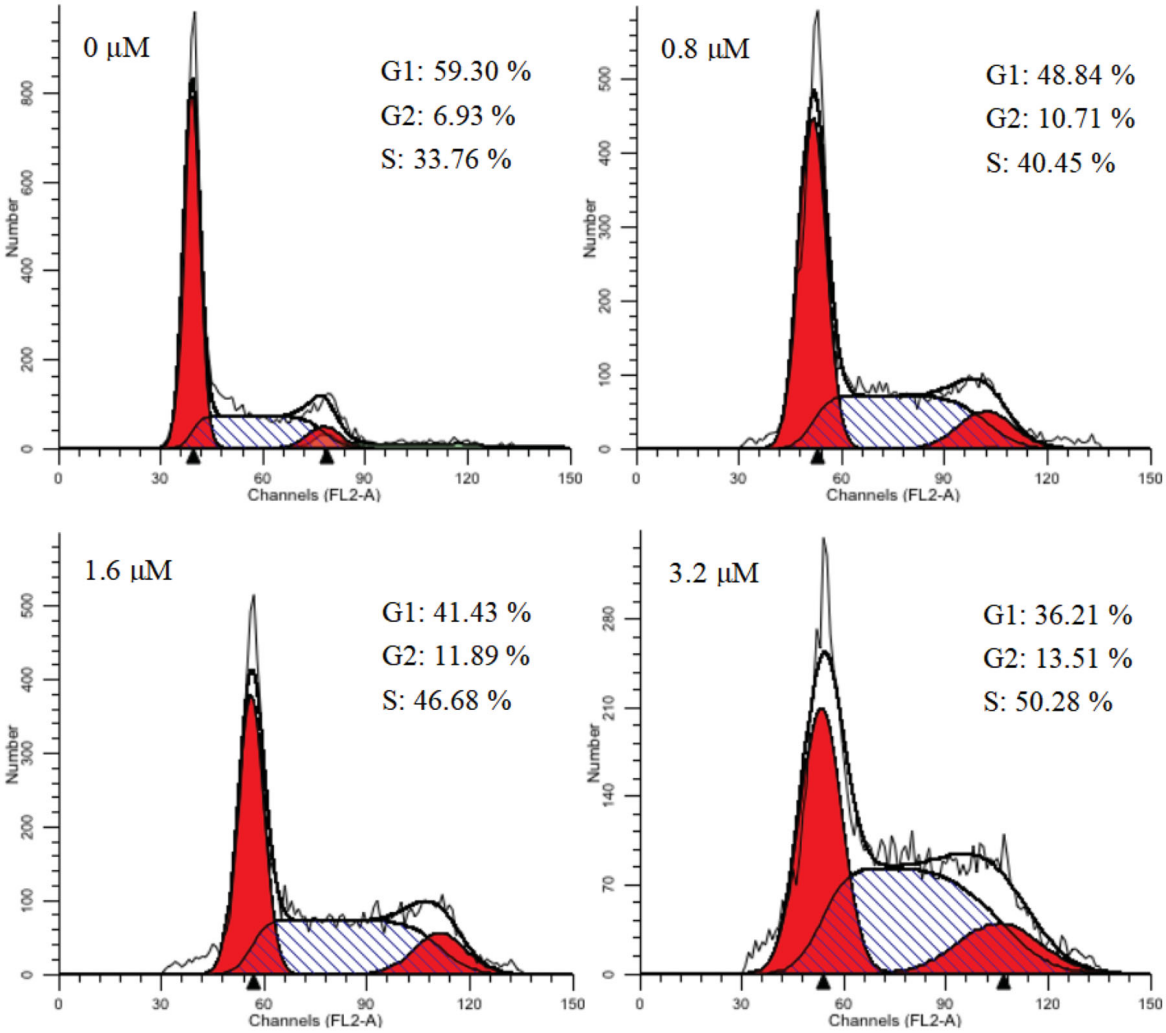
Cell cycle analysis of **15a** (0, 0.8, 1.6, and 3.2 μM) in K562 cells, cells were stained with PI and then cell cycle distribution was analysed by flow cytometry.

Cell cycle refers to the process that is experienced from the end of cell division to the end of the next cell division. Numerous anticancer molecules employ their impact via blocking cell cycle progression, inducing apoptosis, or the merged effects of both^69–71^. To verify the causal relation of cell proliferation inhibition and cell cycle arrest, K562 cells were treated with different concentrations of **15a** (0, 0.8, 1.6 and 3.2 µM). Effects on cell cycle were determined by flow cytometry after propidium iodide (PI) staining. As shown in Figure 4, cells in the S phase increased from 33.76% in the negative control group to 40.45, 46.68 and 50.28% in a concentration-dependent manner. These results revealed that **15a** caused S phase arrest of K562 cells in a concentration-dependent manner.

#### 3.2.5. Morphological analysis by Hoechst 33258 staining

Cell apoptosis shows characteristic morphological changes, including cell shrinkage, chromatin condensation, apoptotic body formation, cytoskeletal disintegration, etc. Among them, the change of nucleus was the most significant one. Hoechst 33258, which stains the cell nucleus, is a membrane permeable dye with blue fluorescence. Live cells with uniformly light blue nuclei can be observed under fluorescence microscope after the treatment with Hoechst 33258. Apoptotic cells have bright blue nuclei on account of karyopyknosis and chromatin condensation; whereas, the nuclei of dead cells cannot be stained^72^.

**Figure 5.**
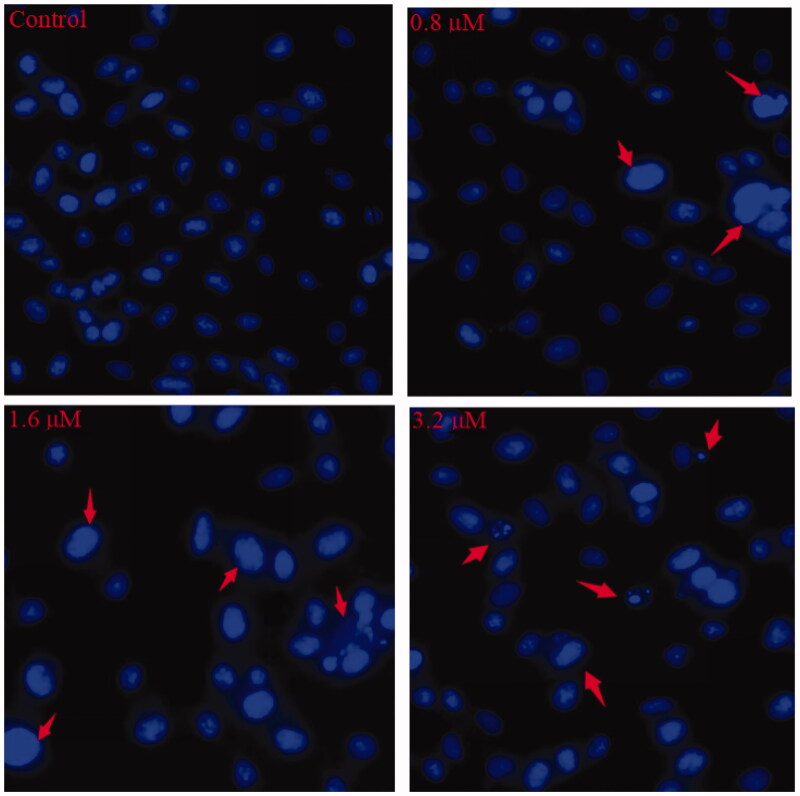
Hoechst staining of **15a-**treated K562 cells. The red arrows point to the cells with obvious morphological changes of apoptosis.

In Figure 5, control cells showed no obvious morphological changes, while K562 cells exposed to 0.8 and 1.6 µM of **15a** exhibited brightly blue fluorescence and revealed typical apoptotic morphology. After the treatment of 3.2 µM of **15a**, the K562 cell membranes were ruptured and the nuclei were fragmented. These results strongly supported the pro-apoptotic effects of **15a**.

#### 3.2.6. Cell apoptosis assay

**Figure 6.**
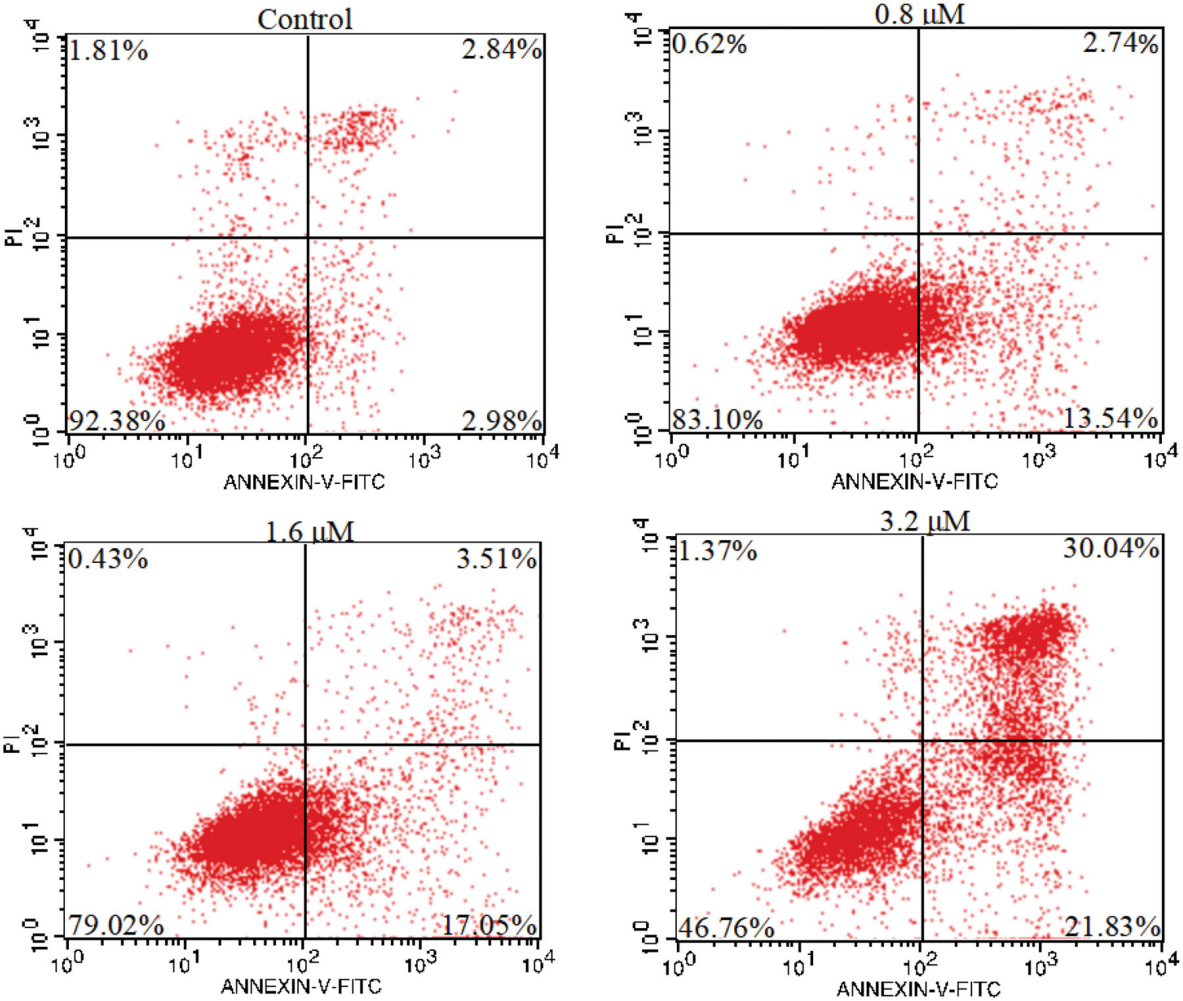
K562 cells were treated with **15a**, stained with annexin V-FITC/PI and analysed by flow cytometry.

NO has been noted in cancer biology that is associated with cancer cell apoptosis^73^. To further verify **15a** induced apoptosis in K562 cells, K562 cells were treated with different concentrations (the same as the cell cycle test) of **15a** for 72 h. Then, cells were harvested and stained with Annexin V-FITC and propidium iodide (PI), and the percentages of apoptotic cells were determined by flow cytometry. As shown in Figure 6, the apoptotic rates of drug-treated cells were positively correlated with the concentrations. At concentrations of 0.8, 1.6 and 3.2 µM, the apoptotic rates were 16.28, 20.56 and 51.87%, respectively, compared with 5.82% in the negative control group, which confirmed that **15a** induced apoptosis in K562 cells.

#### 3.2.7. Mitochondria membrane potential analysis

**Figure 7.**
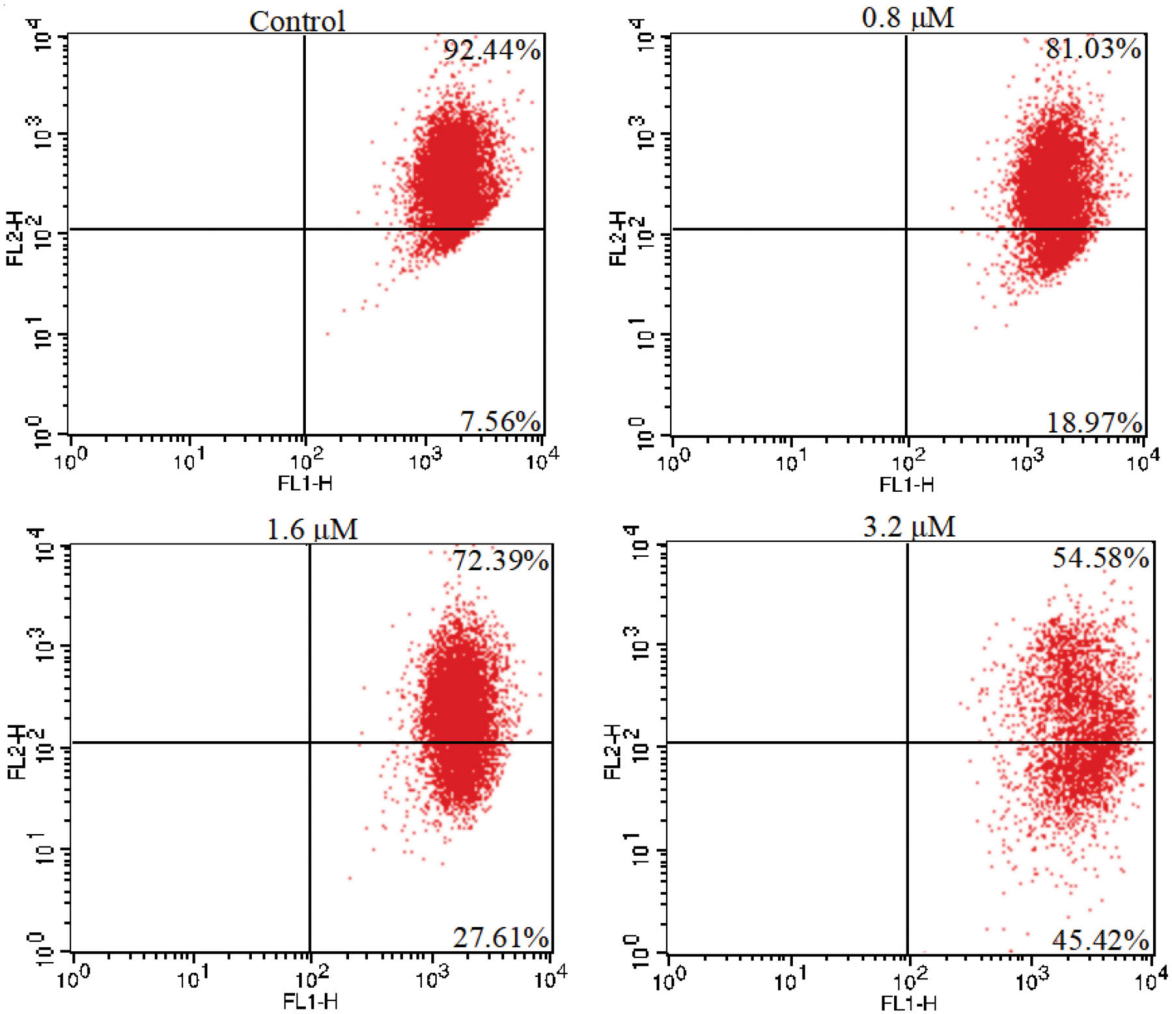
**15a** induced mitochondrial depolarisation in K562 cells.

Mitochondria are the main organelles that produce ATP and play important roles in the process of apoptosis^74–77^. We further examined the mitochondrial membrane potentials of K562 cells treated with **15a** to confirm its pro-apoptotic effects. K562 cells were treated with different concentrations of **15a** for 48 h, then stained with the dye 5,5′,6,6′-tetrachloro-1,1′,3,3′-tetraethylbenzimidazol-caebocyanine (JC-1). The changes of mitochondrial membrane potentials were observed. As shown in Figure 7, the depolarisation of mitochondria increased in a concentration-dependent fashion, indicating that **15a** induced apoptosis through mitochondrial related pathways.

#### 3.2.8. Effects on apoptosis-related protein

**Figure 8.**
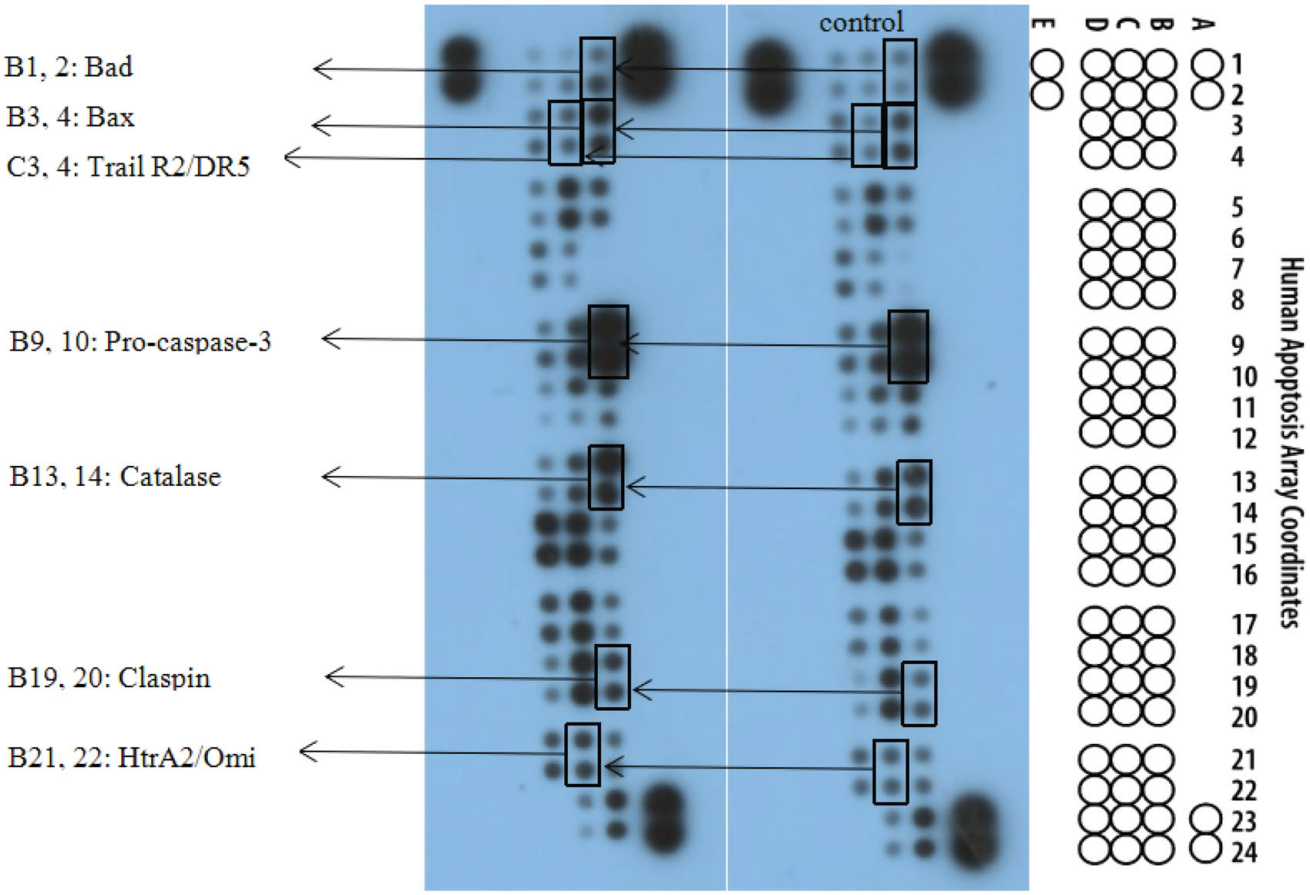
The effects exerted by **15a** on the expression of apoptosis-related proteins using the Human Apoptosis Array kit in K562 cells.

To further investigate the mechanism of action of **15a** in K562 cells, the expression of related apoptotic proteins were tested using the Human Apoptotic Array Kit (Figure 8). The apoptotic pathways of cells mainly include the mitochondrial pathway, the endoplasmic reticulum pathway and the death receptor pathway.

The Bcl-2 family is a major regulator of the mitochondrial apoptotic pathway. Bad and Bax are pro-apoptotic members that form homodimers with their family members, increasing mitochondrial permeability. Caspase-3 is activated by proteolytic processing of procaspase-3 and activates caspases chain, and finally causes apoptosis^78^^,^^79^. From experimental results (Figure 8), increased expression of Bad and Bax was observed after the treatment with **15a** compared to the control.

The death receptor pathway regulates apoptosis mainly through three pathways, including tumour necrosis factor receptor (TNFR), tumour necrosis factor-related apoptosis-inducing ligand (Trail), and factor associated suicide/ligand (Fas/FasL) pathways. The death ligand TRAIL binds to the receptor R2/DR5 on the cell surface and forms death-inducing signalling complex (DISC), thereby activates caspase 8, which in turn causes subsequent cascade reactions^80^^,^^81^. The results also showed upregulated expression of Trail R2/DR5, which were the exogenous pathway results.

HtrA2 is a serine protease located in the mitochondria of eukaryotes. When cells are stimulated, in addition to inducing apoptosis through the mitochondrial pathway, Omi interacts with the antiapoptotic protein HAX-1 (HIS-associated protein X-1) via the caspases-independent pathway, causing HAX-1 degradation, thereby increasing the sensitivity of cells to apoptotic stimuli and inducing apoptosis. P53 also induces HtrA2 phosphorylation by regulating the actin cytoskeleton to counteract cell migration induced by Ras, thereby inhibiting tumour metastasis^82^^,^^83^. Catalase exists in all known animals and regulates the metabolism of reactive oxygen species in the body in a stable state, studies have shown that it inhibits NF-κB activation and promotes apoptosis^84^^,^^85^.

To ensure orderly progression of cell cycle, there are many quality control points known as cell cycle checkpoint proteins. Claspin, as a tumour suppressor, activates checkpoint kinase 1 (Chk1), sensitises cancer cell DNA during the S phase of the cell cycle, produces stress responses, and induces DNA damage and apoptosis^86–89^. The expression of Claspin is increased to achieve the purpose of inhibiting the production of tumour cells.

**Figure 9.**
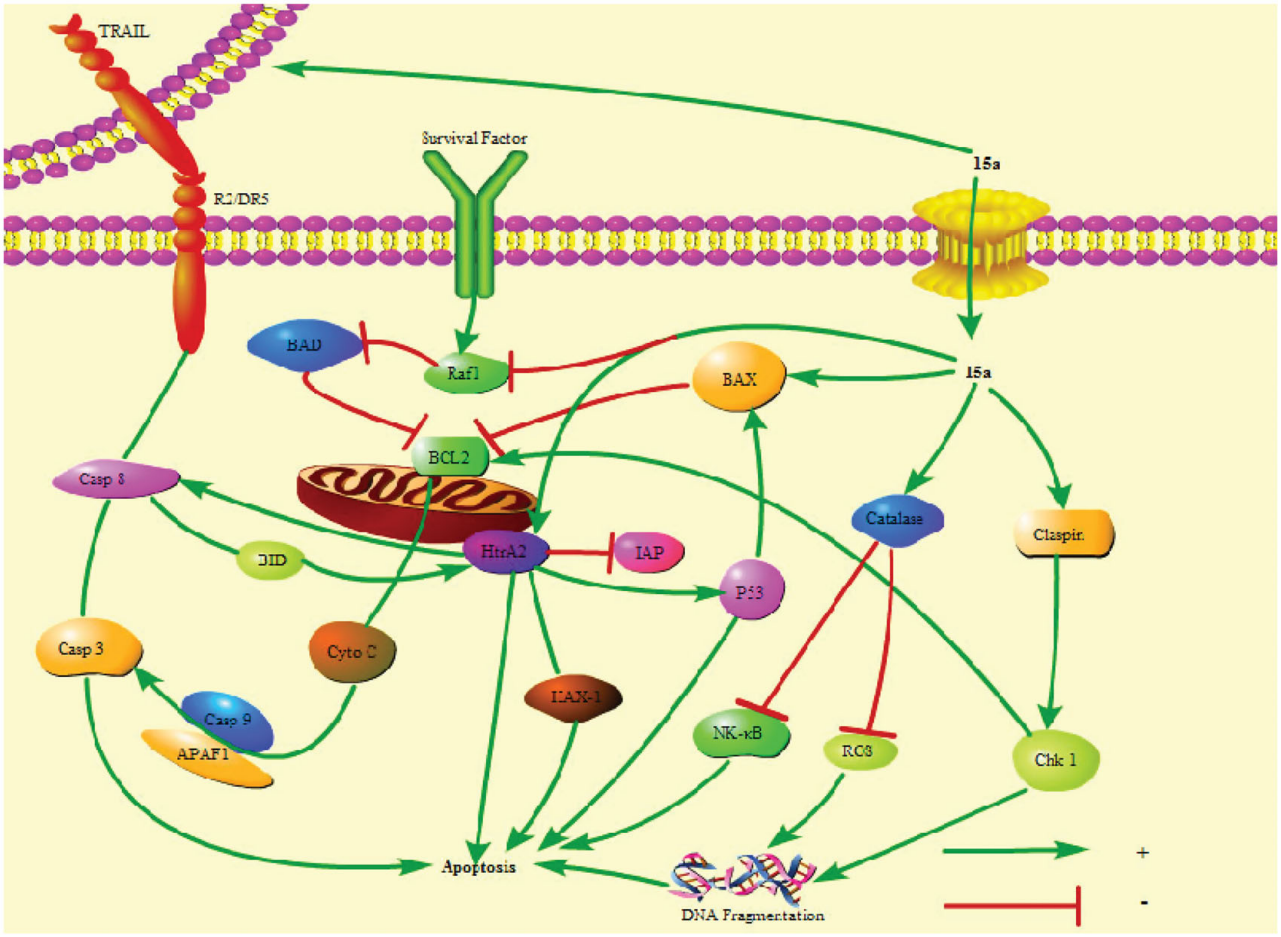
**15a** induced K562 cells death through multiple pathways. Bad and Bax participate in endogenous pathways. Trail R2/DR5 induces cell death through exogenous pathways. HtrA2 kills cells through non-caspase pathway. In addition, catalase promotes apoptosis by inhibiting NF-κB translocation and reactive oxygen content. Claspin indirectly induces DNA damage to kill cells.

The above results indicated that **15a** induced apoptosis of K562 cells by participating in both endogenous and exogenous pathways (Figure 9).

## 4. Conclusion

In conclusion, 16 furoxan-based chromone derivatives were synthesised and tested for their antiproliferative activity. The results indicated that most of the target compounds exhibited stronger antiproliferative activity than the parent compound. **15a** showed the most potent activity with an IC_50_ value of 1.61 µM against K562 cells. To further investigate its mechanism of action, the effects on cell cycle, morphological change, mitochondrial membrane potential and apoptosis-related proteins were evaluated. The results showed that **15a** caused S phase arrest in K562 cells in a concentration-dependent manner. Cells treated with **15a** showed obvious cell membrane rupture and nuclear fragmentation. The apoptotic rate of the treated cells was positively correlated with the concentration. Human apoptosis protein array assay also demonstrated **15a** increased the expression levels of pro-apoptotic Bax, Bad, HtrA2, and Trail R2/DR5. The expression of Catalase and cell cycle blocker Claspin were similarly up-regulated. So, **15a** induced K562 cells death through multiple pathways, including endogenous and exogenous pathways, as well as non caspase pathway. In addition, DNA damage indirectly killed cells. In brief, **15a** as an antitumor drug candidate deserves further investigation.

## Supplementary Material

Supplemental Material
