# Supplementary material for: Antiproliferative chromone derivatives induce K562 cell death through endogenous and exogenous pathways
Source: J Enzyme Inhib Med Chem. 2020 Mar 18;35(1):759–72. doi: 10.1080/14756366.2020.1740696 (PMC7144234; doi:10.1080/14756366.2020.1740696)
Supplement: Supplemental Material [file IENZ_A_1740696_SM3007.pdf]

Antiproliferative chromone derivatives induce K562 cell death through endogenous and exogenous pathways

Runwei Jiao[a,1], Fanxing Xu[b,1], Xiaofang Huang[a], Haonan Li[a], Weiwei Liu[b], Hao Cao[c], Linghe Zang[c], Zhanlin Li[a], Huiming Hua[a], Dahong Li[a,*]

[a]*Key Laboratory of Structure-Based Drug Design & Discovery, Ministry of Education, and School of Traditional Chinese Materia Medica, Shenyang Pharmaceutical University, 103 Wenhua Road, Shenyang 110016, P. R. China*

[b]*Wuya College of Innovation, Shenyang Pharmaceutical University, 103 Wenhua Road, Shenyang 110016, P. R. China*

[c]*School of Life Science and Biopharmaceutics, Shenyang Pharmaceutical University, 103 Wenhua Road, Shenyang 110016, P. R. China*

[*]Corresponding author. E-mail address: lidahong0203@163.com.

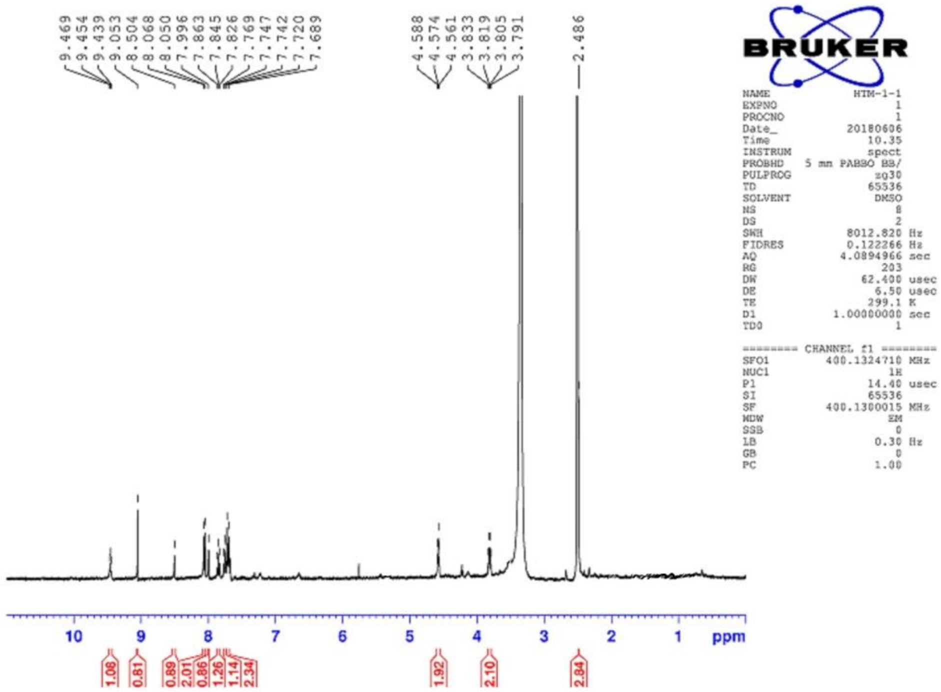

Figure S1. ^{1}H NMR of compound **12a**

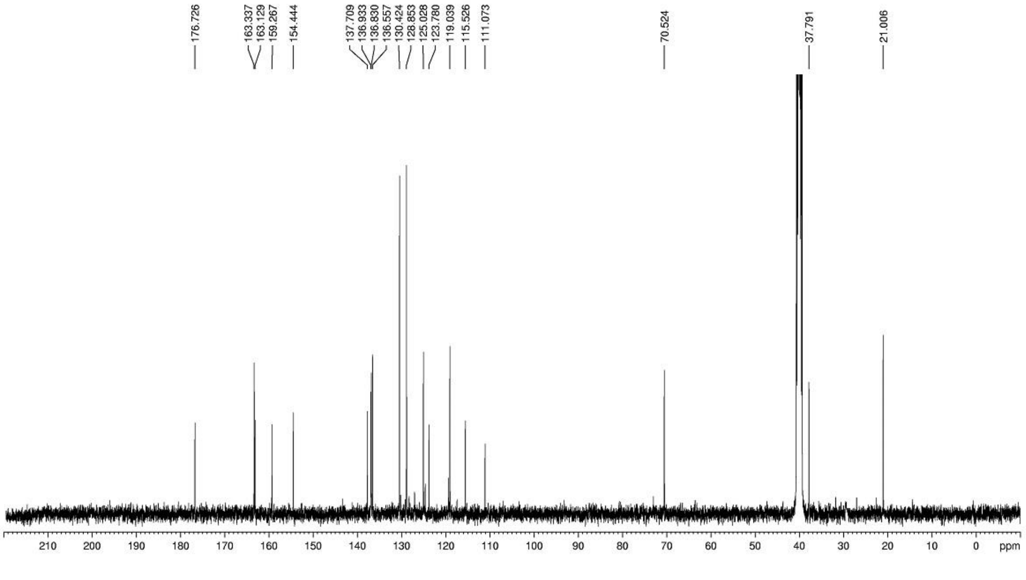

Figure S2. ^{13}C NMR of compound **12a**

Tolerance = 5.0 mDa / DBE: min = -1.5, max = 50.0
Element prediction: Off
Number of isotope peaks used for i-FIT = 3
Monoisotopic Mass, Even Electron Ions
8700 formula(e) evaluated with 92 results within limits (up to 20 closest results for each mass)
Elements Used:
C: 0-50 H: 0-100 N: 0-50 O: 0-100 S: 0-30

Mass	RA	Calc. Mass	mDa	PPM	DBE	Formula	i-FIT	i-FIT Norm	Fit Conf %	C	H	N	O	S
470.0667	100.00	470.0673	-0.6	-1.3	23.5	C30 H16 N O S2	71.4	3.595	2.75	30	16	1	1	2
		470.0665	0.2	0.4	24.5	C29 H12 N O6	71.3	3.481	3.08	29	12	1	6	
		470.0671	-0.4	-0.9	20.5	C22 H12 N7 O4 S	71.2	3.366	3.45	22	12	7	4	1
		470.0667	0.0	0.0	14.5	C22 H20 N3 O3 S3	71.2	3.417	3.28	22	20	3	3	3
		470.0658	0.9	1.9	15.5	C21 H16 N3 O8 S	71.2	3.376	3.42	21	16	3	8	1
		470.0674	-0.7	-1.5	10.5	C15 H20 N9 O S4	71.2	3.413	3.30	15	20	9	1	4
		470.0678	-1.1	-2.3	16.5	C15 H12 N13 O2 S2	71.2	3.378	3.41	15	12	13	2	2
		470.0669	-0.2	-0.4	4.5	C15 H28 N5 S6	71.3	3.518	2.97	15	28	5		6
		470.0660	0.7	1.5	5.5	C14 H24 N5 O5 S4	71.2	3.376	3.42	14	24	5	5	4
		470.0670	-0.3	-0.6	17.5	C14 H8 N13 O7	70.8	3.049	4.74	14	8	13	7	

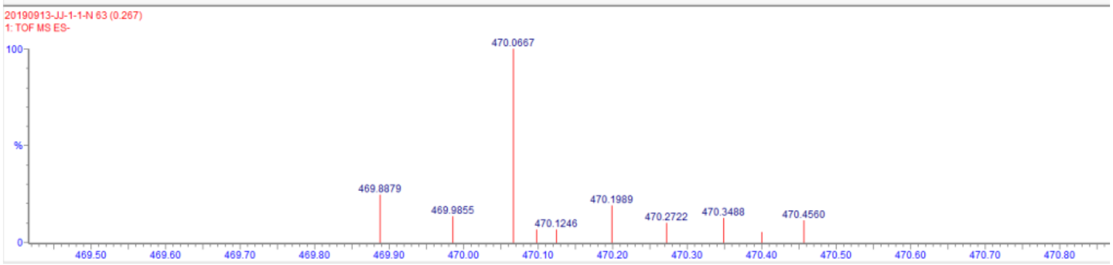

Figure S3. HRMS of compound **12a**

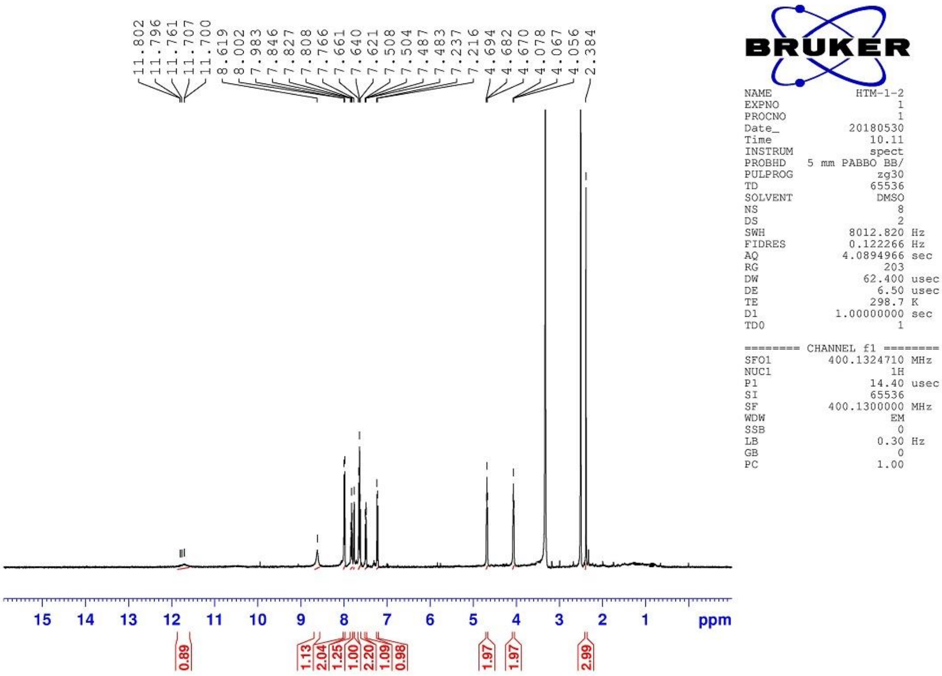

Figure S4. ^{1}H NMR of compound **14a**

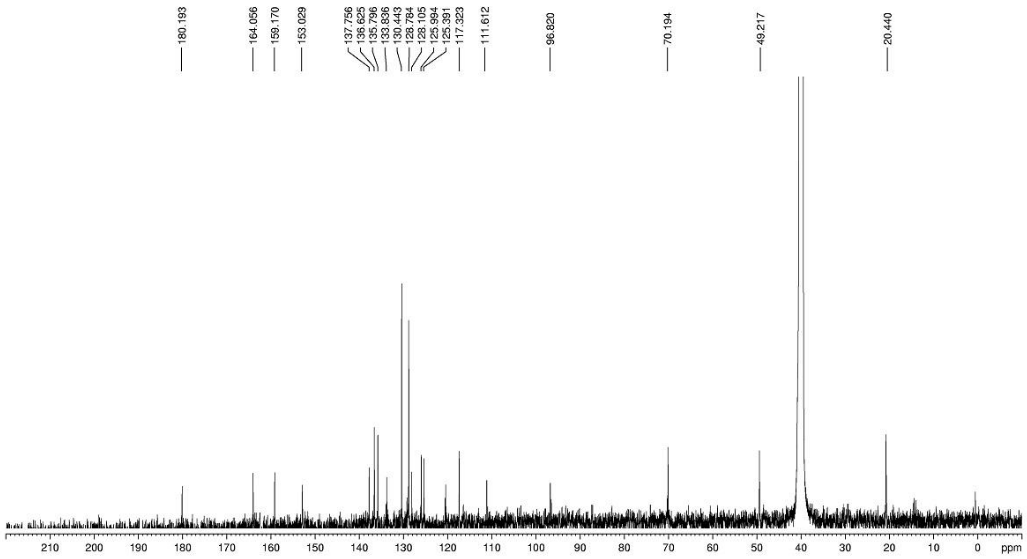

Figure S5. ^{13}C NMR of compound **14a**

Tolerance = 5.0 mDa / DBE: min = -1.5, max = 50.0
Element prediction: Off
Number of isotope peaks used for i-FIT = 3
Monoisotopic Mass, Even Electron Ions
9777 formula(e) evaluated with 101 results within limits (up to 10 closest results for each mass)
Elements Used:
C: 0-50 H: 0-100 N: 0-50 O: 0-100 S: 0-30

Mass	RA	Calc. Mass	mDa	PPM	DBE	Formula	i-FIT	i-FIT Norm	Fit Conf %	C	H	N	O	S
486.0609	100.00	486.0609	0.0	0.0	5.5	C14 H24 N5 O6 S4	21.8	2.496	8.25	14	24	5	6	4
		486.0608	0.1	0.2	2.5	C6 H20 N11 O9 S3	21.5	2.185	11.25	6	20	11	9	3
		486.0607	0.2	0.4	15.5	C21 H16 N3 O9 S	21.8	2.448	8.65	21	16	3	9	1
		486.0612	-0.3	-0.6	8.5	C6 H12 N15 O10 S	21.2	1.844	15.81	6	12	15	10	1
		486.0612	-0.3	-0.6	19.5	C4 N29 S	21.3	1.975	13.88	4		29		1
		486.0605	0.4	0.8	-0.5	C14 H32 N O5 S6	22.0	2.693	6.76	14	32	1	5	6
		486.0613	-0.4	-0.8	-1.5	C15 H36 N S8	22.2	2.850	5.78	15	36	1		8
		486.0605	0.4	0.8	23.5	C11 N23 O2	21.6	2.247	10.57	11		23	2	
		486.0605	0.4	0.8	12.5	C13 H12 N9 O12	21.4	2.108	12.15	13	12	9	12	
		486.0614	-0.5	-1.0	24.5	C29 H12 N O7	22.0	2.673	6.90	29	12	1	7	

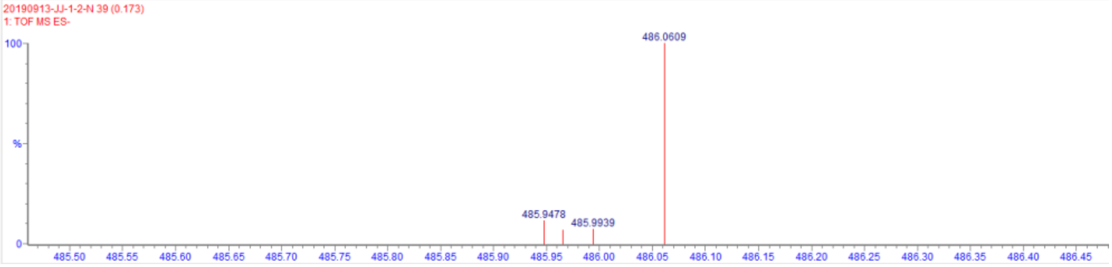

Figure S6. HRMS of compound **14a**

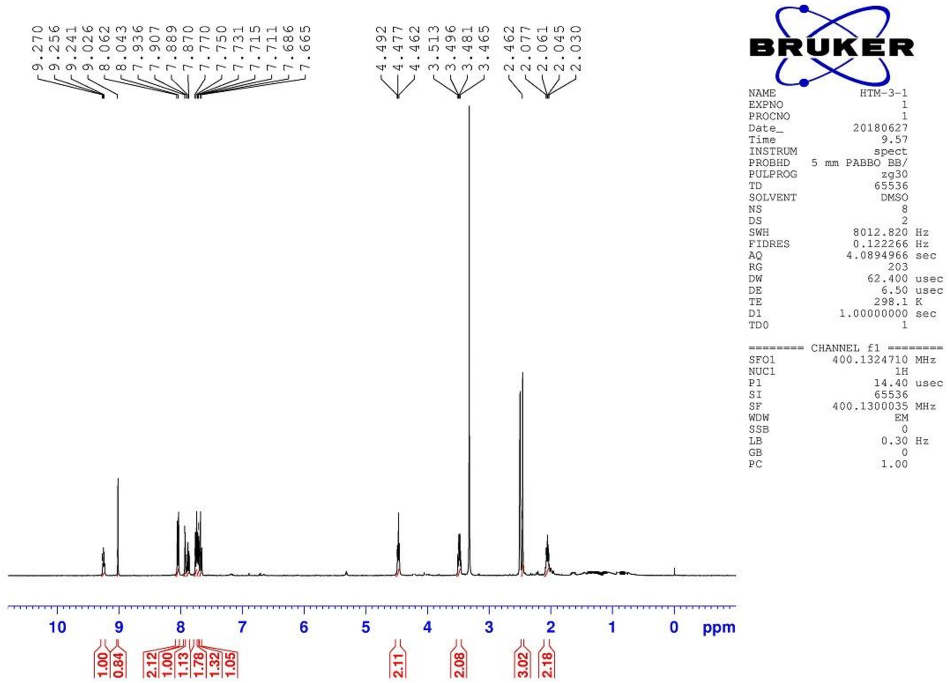

Figure S7. ^{1}H NMR of compound **12b**

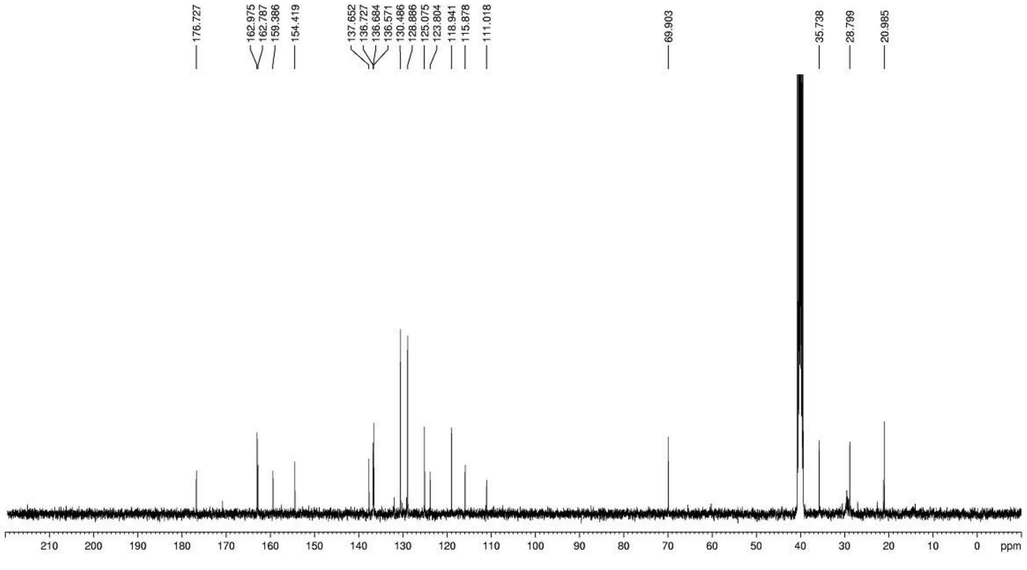

Figure S8. ^{13}C NMR of compound **12b**

Tolerance = 5.0 mDa / DBE: min = -1.5, max = 50.0
Element prediction: Off
Number of isotope peaks used for i-FIT = 3
Monoisotopic Mass, Even Electron Ions
9661 formula(e) evaluated with 96 results within limits (up to 30 closest results for each mass)

Elements Used:
C: 0-50 H: 0-100 N: 0-50 O: 0-100 S: 0-30

Mass	RA	Calc. Mass	mDa	PPM	DBE	Formula	i-FIT	i-FIT Norm	Fit Conf %	C	H	N	O	S
484.0803	100.00	484.0796	0.7	1.4	28.5	C34 H14 N O S	196.5	3.806	2.22	34	14	1	1	1
		484.0808	-0.5	-1.0	30.5	C27 H6 N11	196.4	3.678	2.53	27	6	11		
		484.0794	0.9	1.9	25.5	C26 H10 N7 O4	196.4	3.695	2.48	26	10	7	4	
		484.0790	1.3	2.7	19.5	C26 H18 N3 O3 S2	196.6	3.863	2.10	26	18	3	3	2
		484.0815	-1.2	-2.5	15.5	C22 H18 N3 O8 S	196.4	3.658	2.58	22	18	3	8	1
		484.0796	0.7	1.4	15.5	C19 H18 N9 O S3	196.3	3.598	2.74	19	18	9	1	3
		484.0792	1.1	2.3	9.5	C19 H26 N5 S5	196.5	3.826	2.18	19	26	5		5
		484.0801	0.2	0.4	21.5	C19 H10 N13 O2 S	196.2	3.472	3.11	19	10	13	2	1
		484.0788	1.5	3.1	16.5	C18 H14 N9 O6 S	196.4	3.715	2.44	18	14	9	6	1
		484.0817	-1.4	-2.9	5.5	C15 H26 N5 O5 S4	196.5	3.765	2.32	15	26	5	5	4

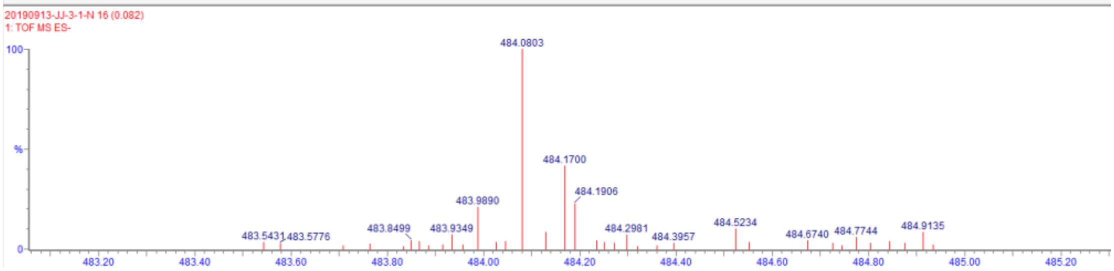

Figure S9. HRMS of compound **12b**

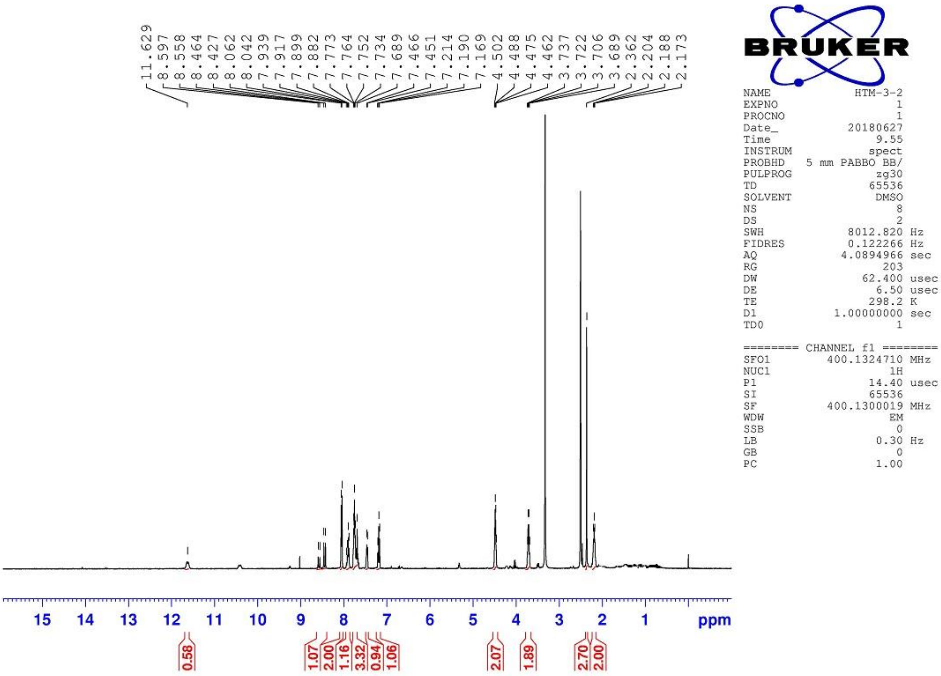

Figure S10. ^{1}H NMR of compound **14b**

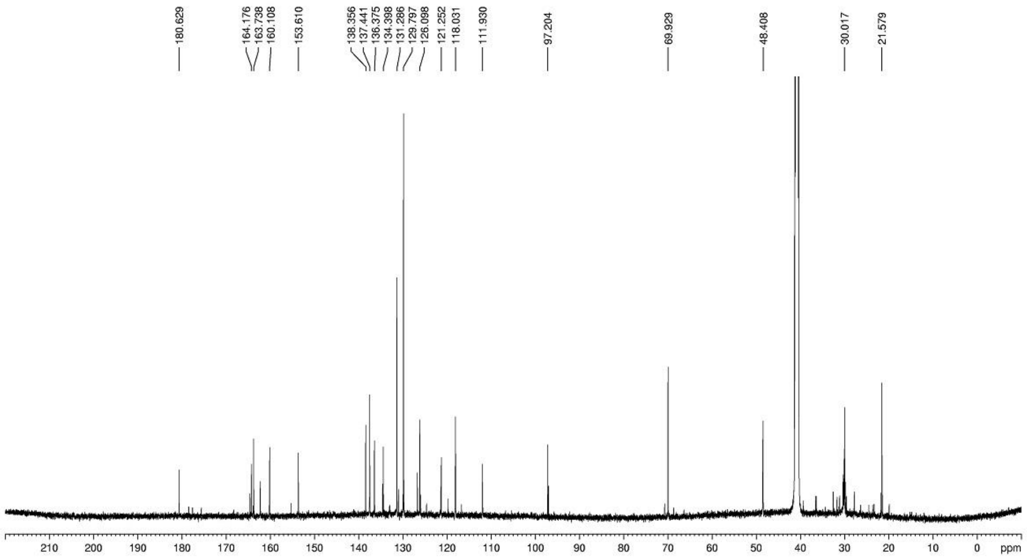

Figure S11. ^{13}C NMR of compound **14b**

Tolerance = 5.0 mDa / DBE: min = -1.5, max = 50.0
Element prediction: Off
Number of isotope peaks used for i-FIT = 3
Monoisotopic Mass, Even Electron Ions
10822 formula(e) evaluated with 106 results within limits (up to 50 closest results for each mass)
Elements Used:
C: 0-50 H: 0-100 N: 0-50 O: 0-100 S: 0-30

Mass	RA	Calc. Mass	mDa	PPM	DBE	Formula	i-FIT	i-FIT Norm	Fit Conf %	C	H	N	O	S
500.0783	100.00	500.0761	2.2	4.4	-0.5	C15 H34 N O5 S6	78.7	4.856	0.78	15	34	1	5	6
		500.0806	-2.3	-4.6	9.5	C20 H26 N3 O4 S4	78.7	4.843	0.79	20	26	3	4	4
		500.0804	-2.1	-4.2	19.5	C27 H18 N O7 S	78.6	4.730	0.88	27	18	1	7	1
		500.0770	1.3	2.6	-1.5	C16 H38 N S8	78.6	4.669	0.94	16	38	1		8
		500.0764	1.9	3.8	15.5	C22 H18 N3 O9 S	78.4	4.527	1.08	22	18	3	9	1
		500.0800	-1.7	-3.4	0.5	C12 H30 N5 O6 S5	78.4	4.502	1.11	12	30	5	6	5
		500.0802	-1.9	-3.8	27.5	C17 H2 N21	78.4	4.502	1.11	17	2	21		
		500.0804	-2.1	-4.2	6.5	C12 H22 N9 O7 S3	78.4	4.495	1.12	12	22	9	7	3
		500.0770	1.3	2.6	24.5	C30 H14 N O7	78.4	4.493	1.12	30	14	1	7	
		500.0766	1.7	3.4	5.5	C15 H26 N5 O6 S4	78.4	4.484	1.13	15	26	5	6	4

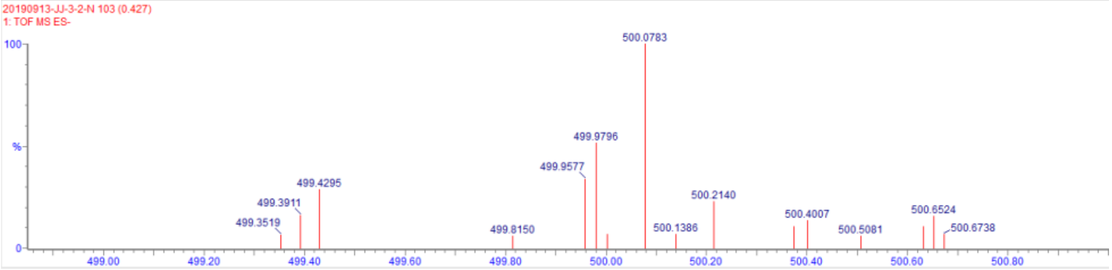

Figure S12. HRMS of compound **14b**

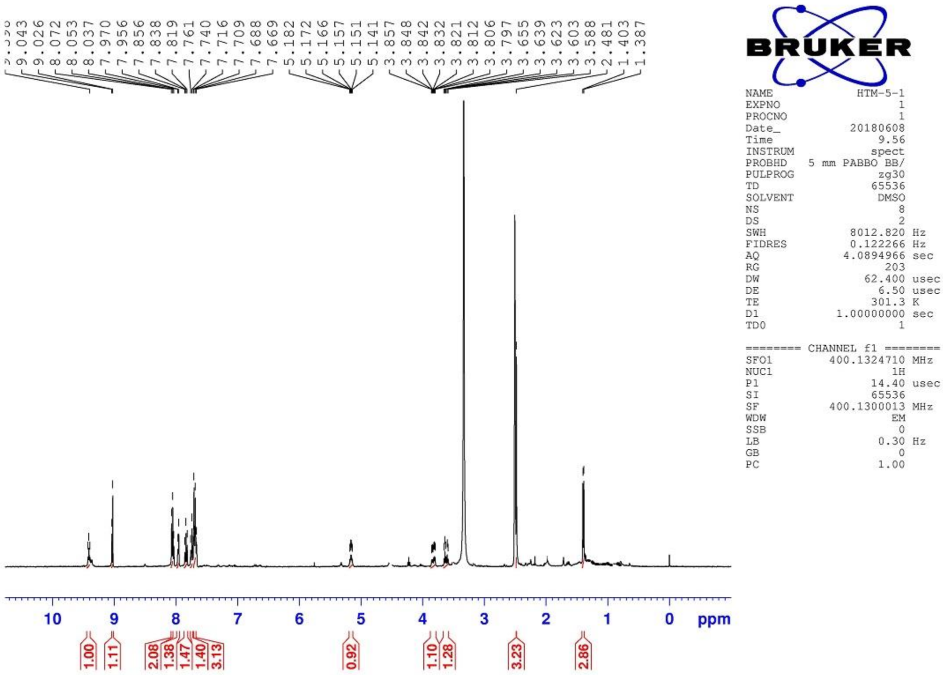

Figure S13. ^{1}H NMR of compound **12c**

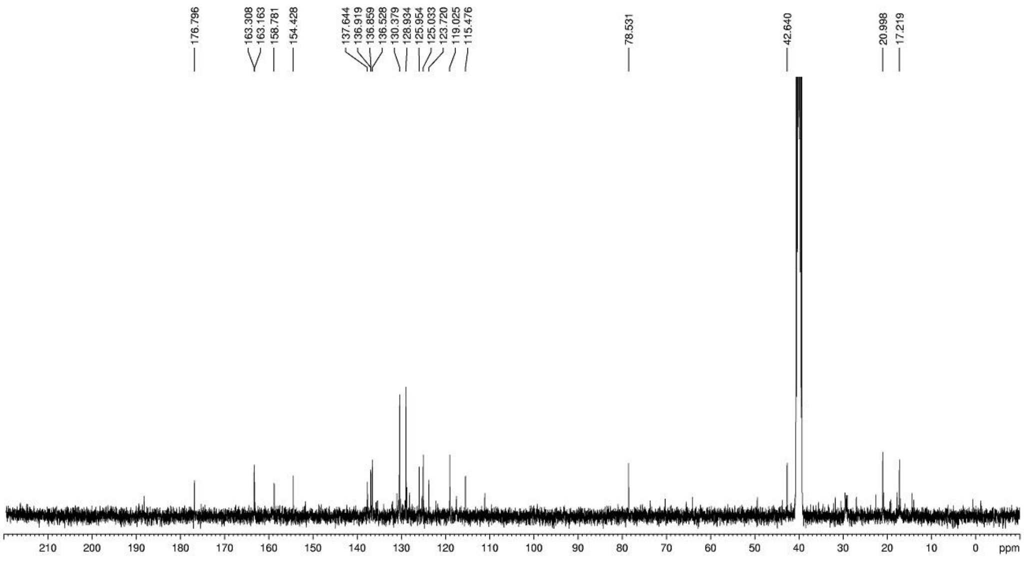

Figure S14. ^{13}C NMR of compound **12c**

Tolerance = 5.0 mDa / DBE: min = -1.5, max = 50.0
Element prediction: Off
Number of isotope peaks used for i-FIT = 3
Monoisotopic Mass, Even Electron Ions
9661 formula(e) evaluated with 96 results within limits (up to 50 closest results for each mass)
Elements Used:
C: 0-50 H: 0-100 N: 0-50 O: 0-100 S: 0-30

Mass	RA	Calc. Mass	mDa	PPM	DBE	Formula	i-FIT	i-FIT Norm	Fit Conf %	C	H	N	O	S
484.0800	100.00	484.0796	0.4	0.8	28.5	C34 H14 N O S	117.3	4.118	1.63	34	14	1	1	1
		484.0821	-2.1	-4.3	24.5	C30 H14 N O6	117.8	4.590	1.02	30	14	1	6	
		484.0808	-0.8	-1.7	30.5	C27 H6 N11	117.3	4.088	1.68	27	6	11		
		484.0794	0.6	1.2	25.5	C26 H10 N7 O4	117.2	3.992	1.85	26	10	7	4	
		484.0790	1.0	2.1	19.5	C26 H18 N3 O3 S2	117.3	4.123	1.62	26	18	3	3	2
		484.0781	1.9	3.9	20.5	C25 H14 N3 O8	117.6	4.361	1.28	25	14	3	8	
		484.0823	-2.3	-4.8	14.5	C23 H22 N3 O3 S3	117.9	4.647	0.96	23	22	3	3	3
		484.0815	-1.5	-3.1	15.5	C22 H18 N3 O8 S	117.3	4.122	1.62	22	18	3	8	1
		484.0801	-0.1	-0.2	21.5	C19 H10 N13 O2 S	117.0	3.827	2.18	19	10	13	2	1
		484.0792	0.8	1.7	9.5	C19 H26 N5 S5	117.3	4.101	1.66	19	26	5		5

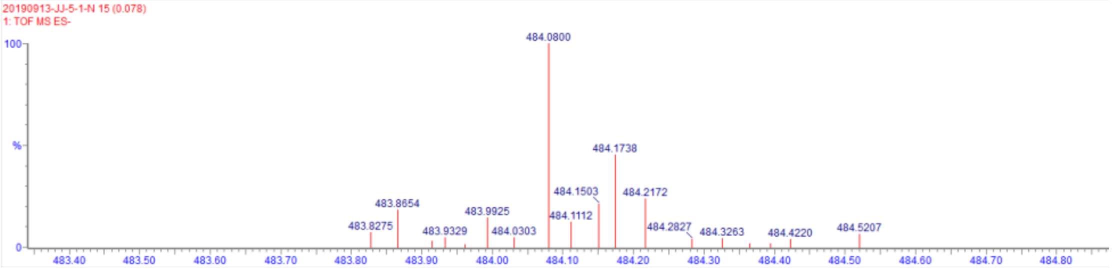

Figure S15. HRMS of compound **12c**

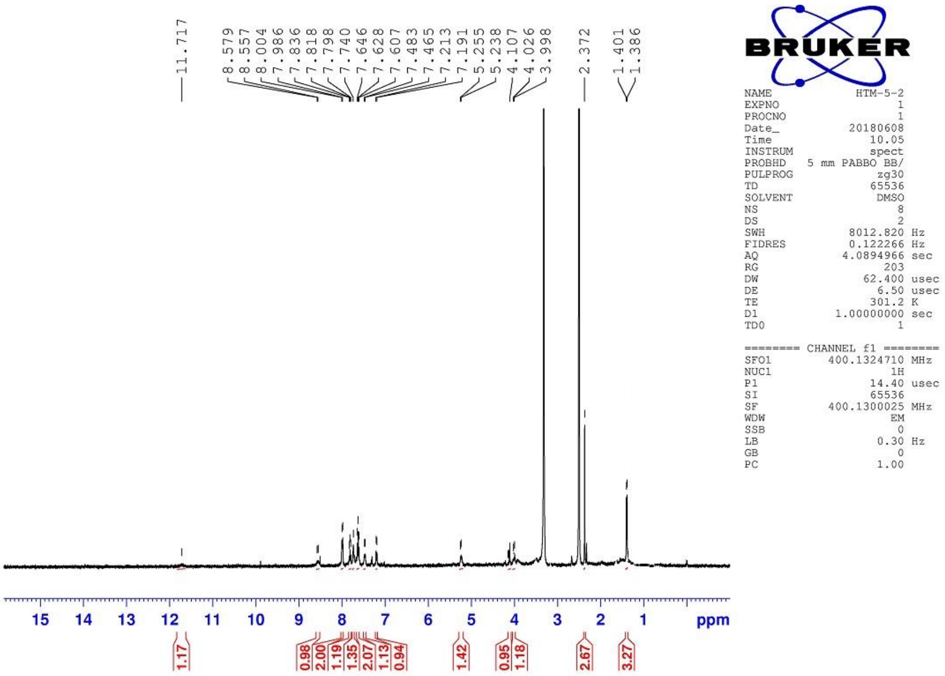

Figure S16. ^{1}H NMR of compound **14c**

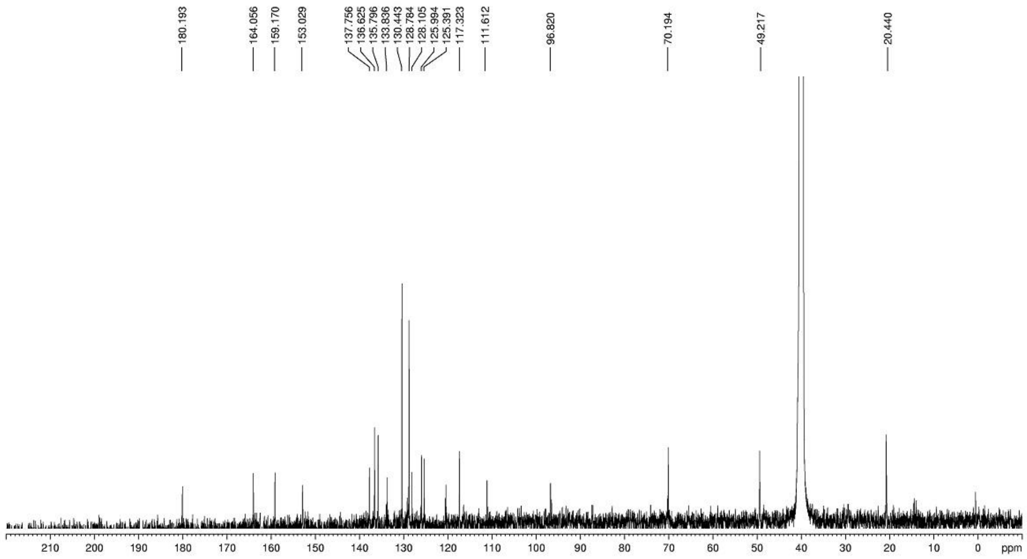

Figure S17. ^{13}C NMR of compound **14c**

Tolerance = 5.0 mDa / DBE: min = -1.5, max = 50.0
Element prediction: Off
Number of isotope peaks used for i-FIT = 3
Monoisotopic Mass, Even Electron Ions
10823 formula(e) evaluated with 106 results within limits (up to 50 closest results for each mass)
Elements Used:
C: 0-50 H: 0-100 N: 0-50 O: 0-100 S: 0-30

Mass	RA	Calc. Mass	mDa	PPM	DBE	Formula	i-FIT	i-FIT Norm	Fit Conf %	C	H	N	O	S
500.0776	100.00	500.0784	-0.8	-1.6	29.5	C31 H10 N5 O3	97.9	4.323	1.33	31	10	5	3	
		500.0779	-0.3	-0.6	23.5	C31 H18 N O2 S2	97.9	4.269	1.40	31	18	1	2	2
		500.0770	0.6	1.2	24.5	C30 H14 N O7	97.8	4.237	1.45	30	14	1	7	
		500.0752	2.4	4.8	24.5	C27 H14 N7 S2	98.5	4.897	0.75	27	14	7		2
		500.0757	1.9	3.8	30.5	C27 H6 N11 O	98.2	4.656	0.95	27	6	11	1	
		500.0790	-1.4	-2.8	25.5	C24 H10 N11 O S	98.0	4.401	1.23	24	10	11	1	1
		500.0786	-1.0	-2.0	19.5	C24 H18 N7 S3	97.9	4.312	1.34	24	18	7		3
		500.0777	-0.1	-0.2	20.5	C23 H14 N7 O5 S	97.7	4.074	1.70	23	14	7	5	1
		500.0772	0.4	0.8	14.5	C23 H22 N3 O4 S3	97.7	4.158	1.56	23	22	3	4	3
		500.0764	1.2	2.4	15.5	C22 H18 N3 O9 S	97.8	4.202	1.50	22	18	3	9	1

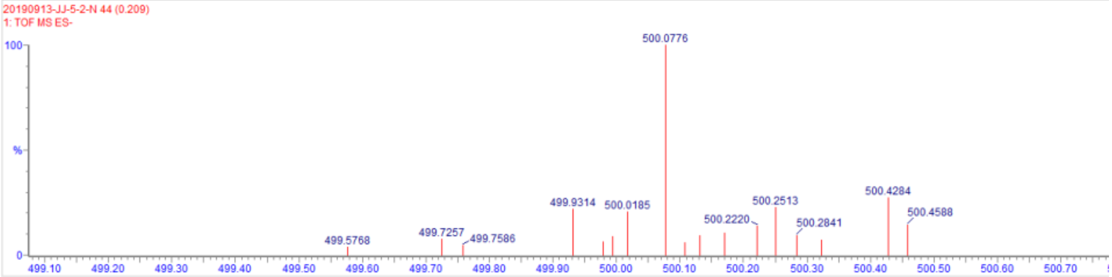

Figure S18. HRMS of compound **14c**

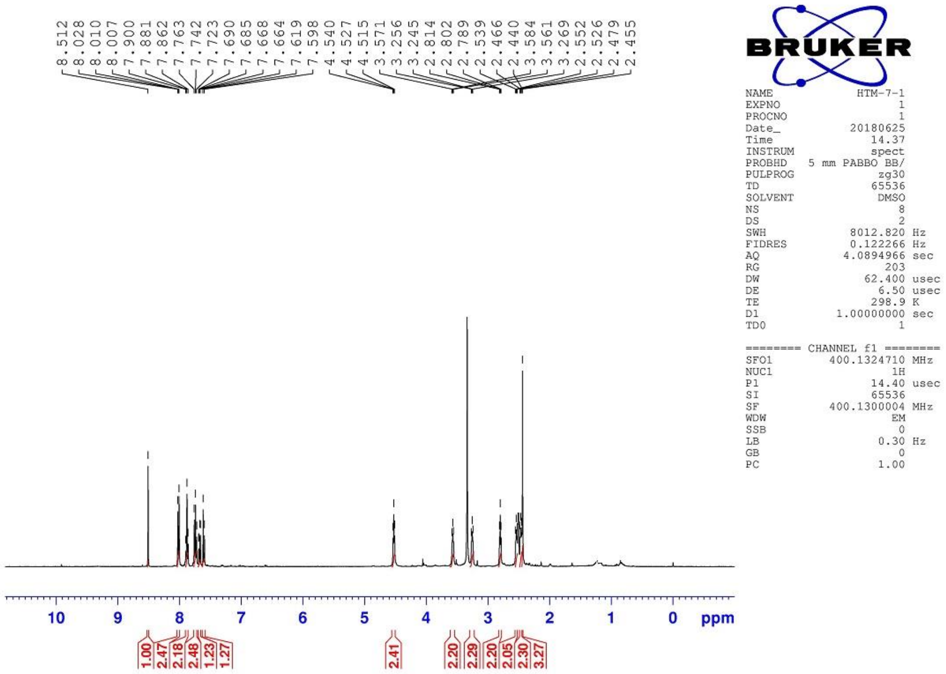

Figure S19. ^{1}H NMR of compound **12d**

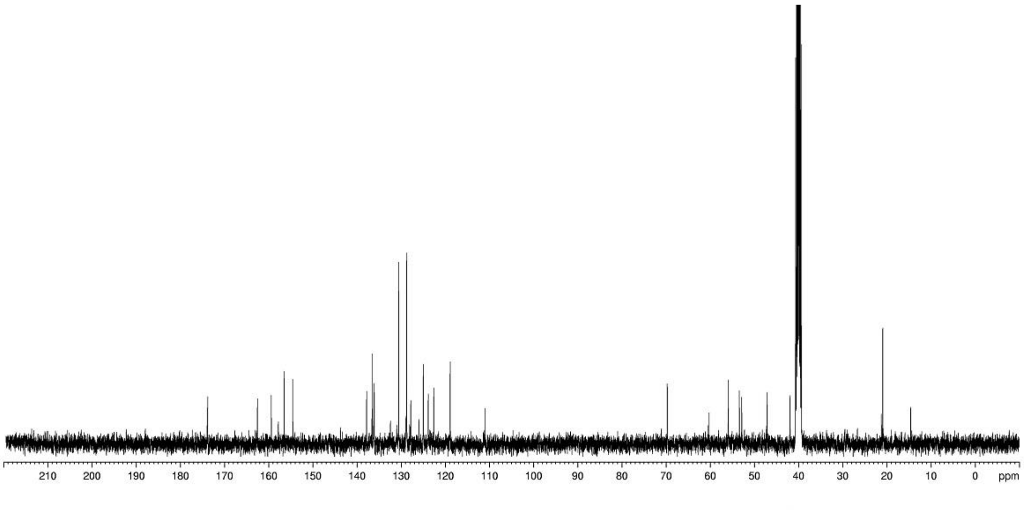

Figure S20. ^{13}C NMR of compound **12d**

Tolerance = 5.0 mDa / DBE: min = -1.5, max = 50.0
Element prediction: Off
Number of isotope peaks used for i-FIT = 3
Monoisotopic Mass, Even Electron Ions
14369 formula(e) evaluated with 120 results within limits (up to 20 closest results for each mass)
Elements Used:
C: 0-50 H: 0-100 N: 0-50 O: 0-100 S: 0-30

Mass	RA	Calc. Mass	mDa	PPM	DBE	Formula	i-FIT	i-FIT Norm	Fit Conf %	C	H	N	O	S
539.1229	100.00	539.1235	-0.6	-1.1	11.5	H11 N32 O S2	477.2	6.855	0.11		11	32	1	2
		539.1228	0.1	0.2	15.5	C7 H11 N26 O3 S	477.2	6.835	0.11	7	11	26	3	1
		539.1235	-0.6	-1.1	0.5	C2 H23 N18 O11 S2	476.9	6.522	0.15	2	23	18	11	2
		539.1228	0.1	0.2	4.5	C9 H23 N12 O13 S	476.7	6.328	0.18	9	23	12	13	1
		539.1226	0.3	0.6	1.5	C H19 N18 O16	476.6	6.226	0.20	1	19	18	16	
		539.1230	-0.1	-0.2	18.5	C15 H15 N20 S2	476.5	6.190	0.21	15	15	20		2
		539.1223	0.6	1.1	22.5	C22 H15 N14 O2 S	476.5	6.186	0.21	22	15	14	2	1
		539.1223	0.6	1.1	9.5	C7 H19 N22 O2 S3	476.2	5.832	0.29	7	19	22	2	3
		539.1237	-0.8	-1.5	16.5	C25 H23 N4 O8 S	476.1	5.789	0.31	25	23	4	8	1
		539.1230	-0.1	-0.2	7.5	C17 H27 N6 O10 S2	476.1	5.713	0.33	17	27	6	10	2

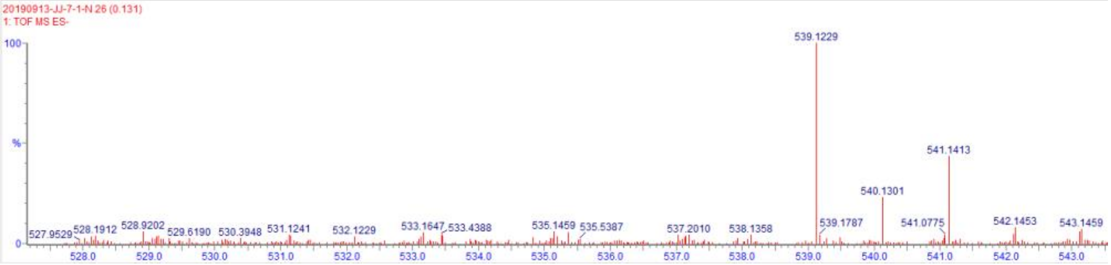

Figure S21. HRMS of compound **12d**

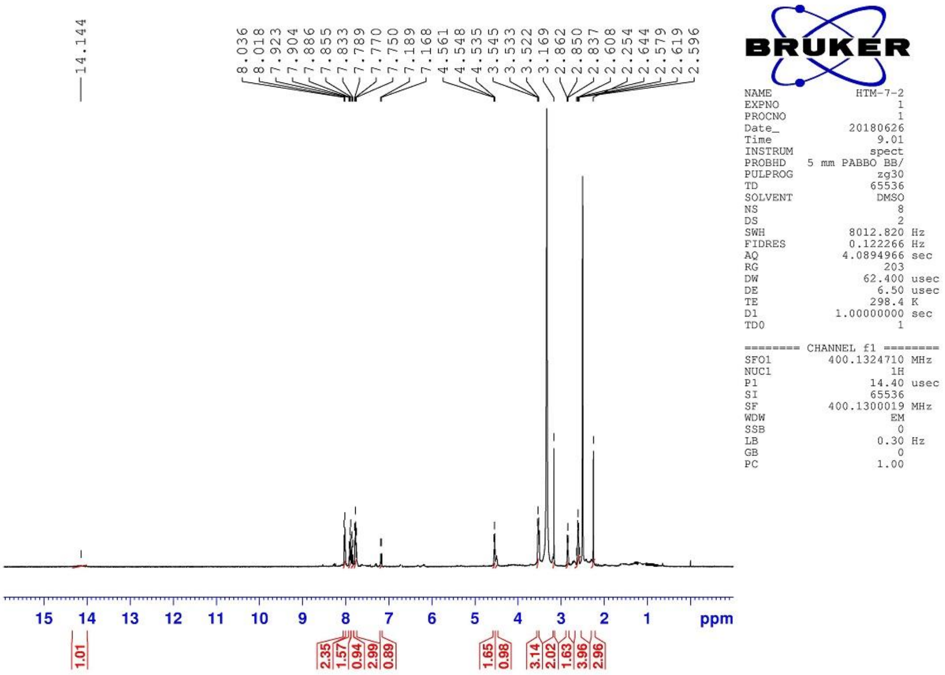

Figure S22. ^{1}H NMR of compound **14d**

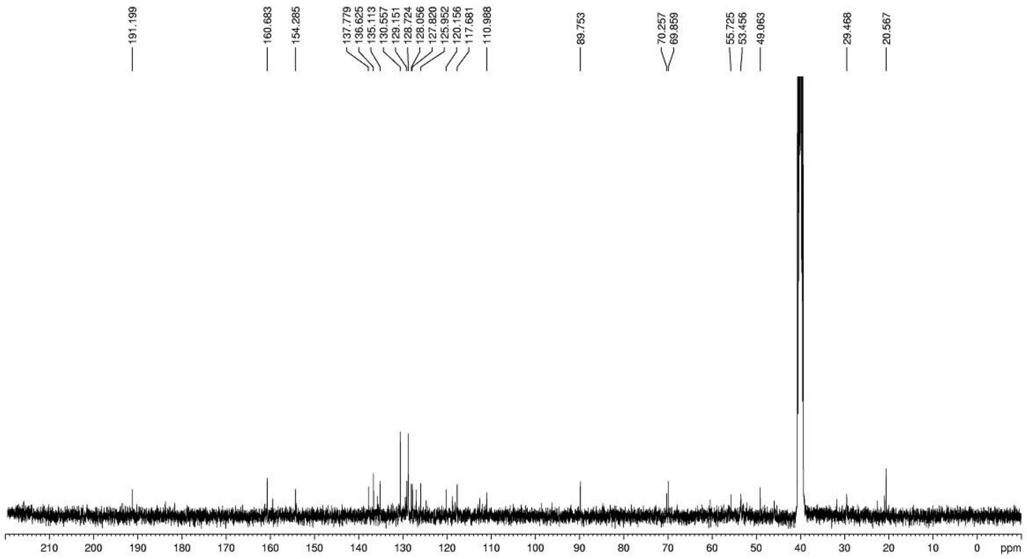

Figure S23. ^{13}C NMR of compound **14d**

Tolerance = 5.0 mDa / DBE: min = -1.5, max = 50.0
Element prediction: Off
Number of isotope peaks used for i-FIT = 3
Monoisotopic Mass, Even Electron Ions
15930 formula(e) evaluated with 137 results within limits (up to 50 closest results for each mass)
Elements Used:
C: 0-50 H: 0-100 N: 0-50 O: 0-100 S: 0-30

Mass	RA	Calc. Mass	mDa	PPM	DBE	Formula	i-FIT	i-FIT Norm	Fit Conf %	C	H	N	O	S
555.1171	100.00	555.1174	-0.3	-0.5	38.5	C45 H15	24.7	4.343	1.30	45	15			
		555.1167	0.4	0.7	29.5	C37 H19 N2 O2 S	24.6	4.255	1.42	37	19	2	2	1
		555.1179	-0.8	-1.4	31.5	C30 H11 N12 O	24.6	4.222	1.47	30	11	12	1	
		555.1174	-0.3	-0.5	25.5	C30 H19 N8 S2	24.6	4.170	1.54	30	19	8		2
		555.1156	1.5	2.7	14.5	C29 H31 O3 S4	24.9	4.477	1.14	29	31		3	4
		555.1161	1.0	1.8	20.5	C29 H23 N4 O4 S2	24.6	4.261	1.41	29	23	4	4	2
		555.1165	0.6	1.1	26.5	C29 H15 N8 O5	24.5	4.150	1.58	29	15	8	5	
		555.1181	-1.0	-1.8	10.5	C25 H31 O8 S3	24.6	4.184	1.52	25	31		8	3
		555.1186	-1.5	-2.7	16.5	C25 H23 N4 O9 S	24.7	4.274	1.39	25	23	4	9	1
		555.1172	-0.1	-0.2	11.5	C24 H27 O13 S	24.3	3.937	1.95	24	27		13	1

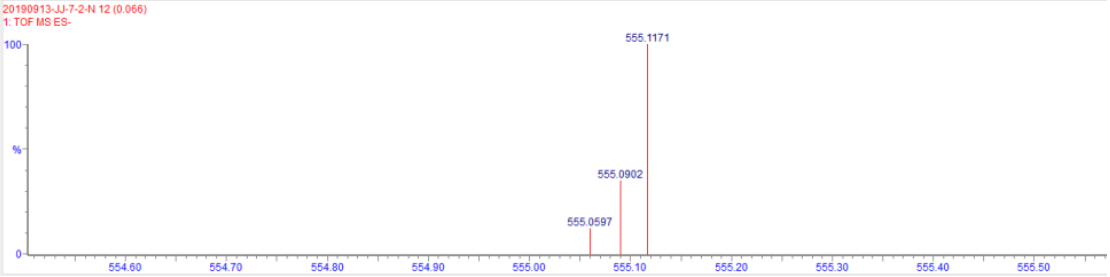

Figure S24. HRMS of compound **14d**

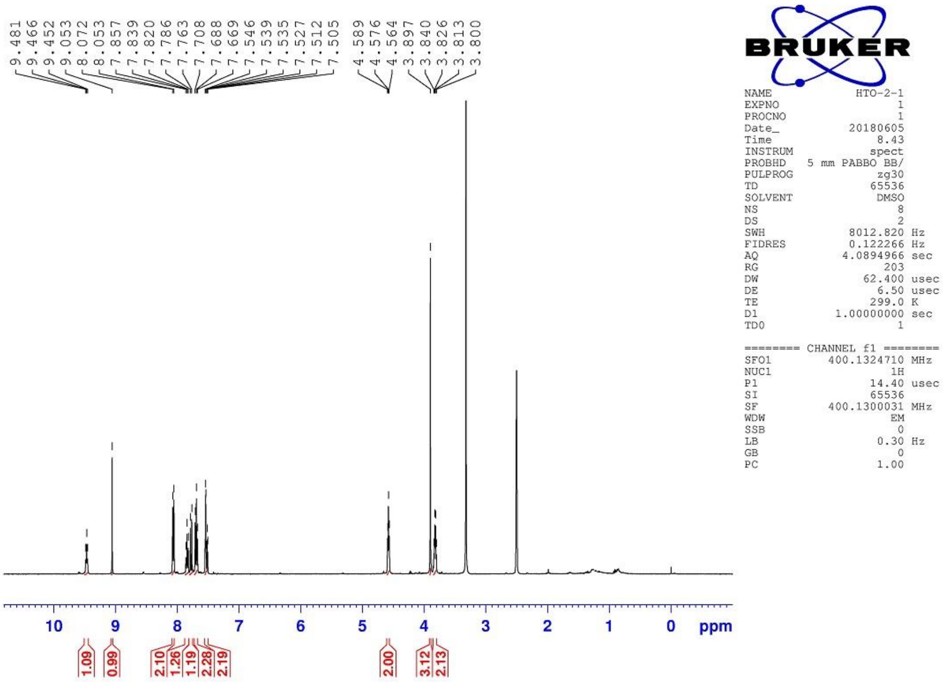

Figure S25. ^{1}H NMR of compound **13a**

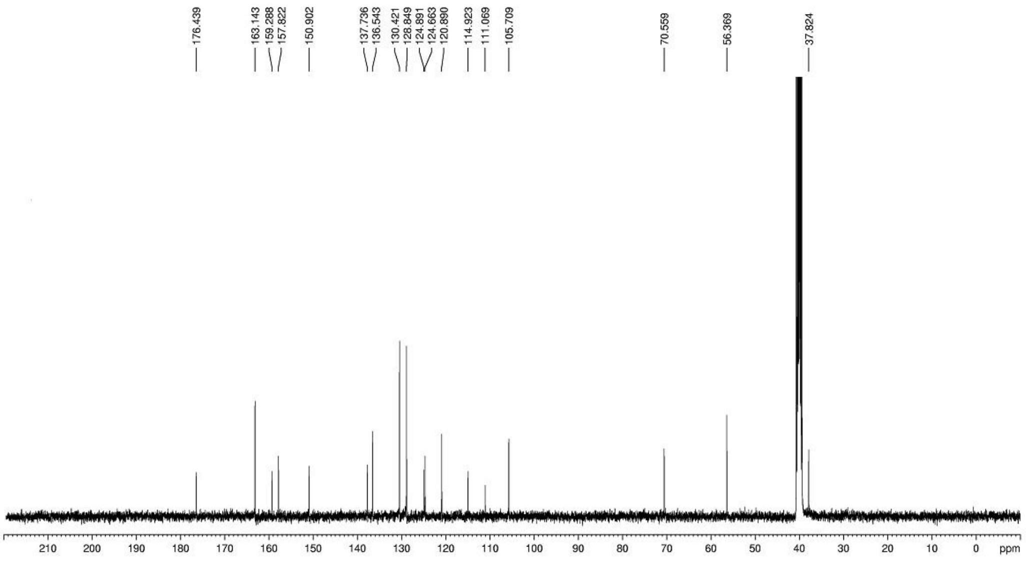

Figure S26. ^{13}C NMR of compound **13a**

Tolerance = 5.0 mDa / DBE: min = -1.5, max = 50.0
Element prediction: Off
Number of isotope peaks used for i-FIT = 3
Monoisotopic Mass, Even Electron Ions
9777 formula(e) evaluated with 101 results within limits (up to 10 closest results for each mass)
Elements Used:
C: 0-50 H: 0-100 N: 0-50 O: 0-100 S: 0-30

Mass	RA	Calc. Mass	mDa	PPM	DBE	Formula	i-FIT	i-FIT Norm	Fit Conf %	C	H	N	O	S
486.0609	100.00	486.0609	0.0	0.0	5.5	C14 H24 N5 O6 S4	82.9	2.450	8.63	14	24	5	6	4
		486.0608	0.1	0.2	2.5	C6 H20 N11 O9 S3	82.6	2.188	11.21	6	20	11	9	3
		486.0607	0.2	0.4	15.5	C21 H16 N3 O9 S	82.8	2.409	8.99	21	16	3	9	1
		486.0612	-0.3	-0.6	8.5	C6 H12 N15 O10 S	82.3	1.924	14.60	6	12	15	10	1
		486.0612	-0.3	-0.6	19.5	C4 N29 S	82.4	2.023	13.23	4		29		1
		486.0605	0.4	0.8	-0.5	C14 H32 N O5 S6	83.0	2.630	7.20	14	32	1	5	6
		486.0613	-0.4	-0.8	-1.5	C15 H36 N S8	83.2	2.773	6.25	15	36	1		8
		486.0605	0.4	0.8	23.5	C11 N23 O2	82.7	2.241	10.63	11		23	2	
		486.0605	0.4	0.8	12.5	C13 H12 N9 O12	82.5	2.128	11.91	13	12	9	12	
		486.0614	-0.5	-1.0	24.5	C29 H12 N O7	83.0	2.612	7.34	29	12	1	7	

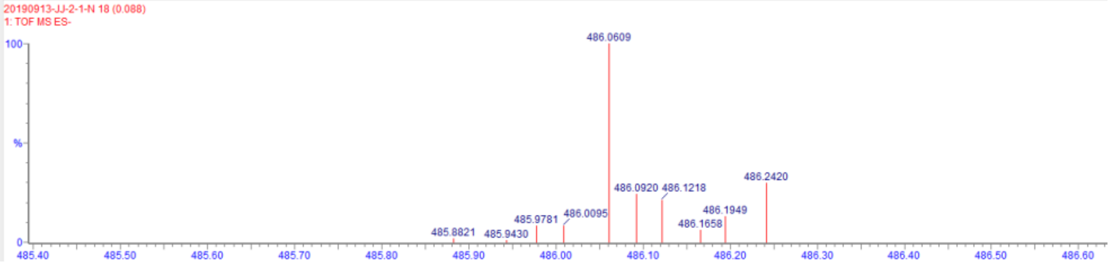

Figure S27. HRMS of compound **13a**

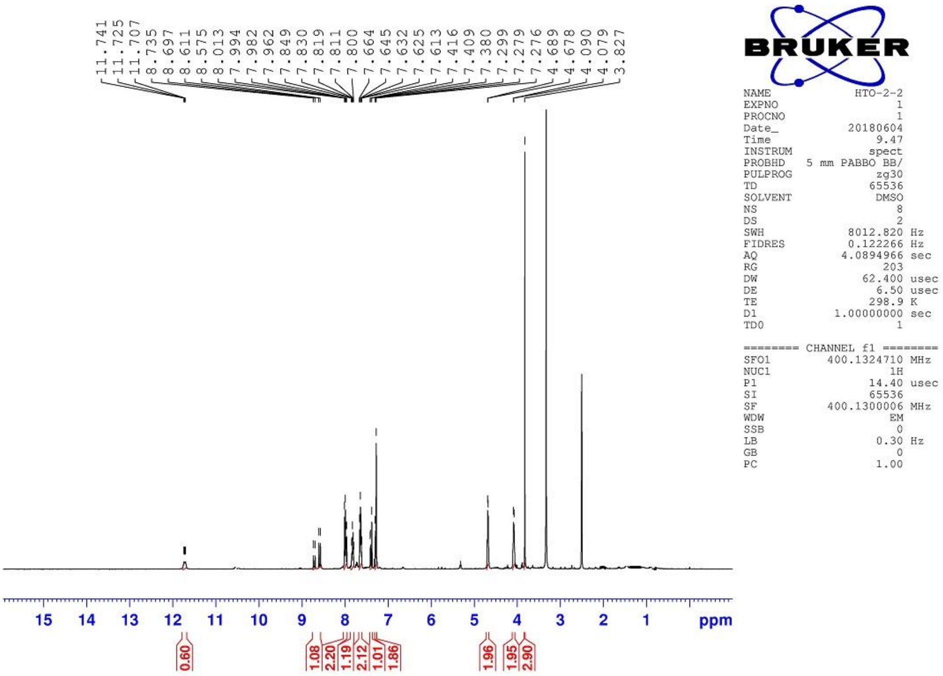

Figure S28. ^{1}H NMR of compound **15a**

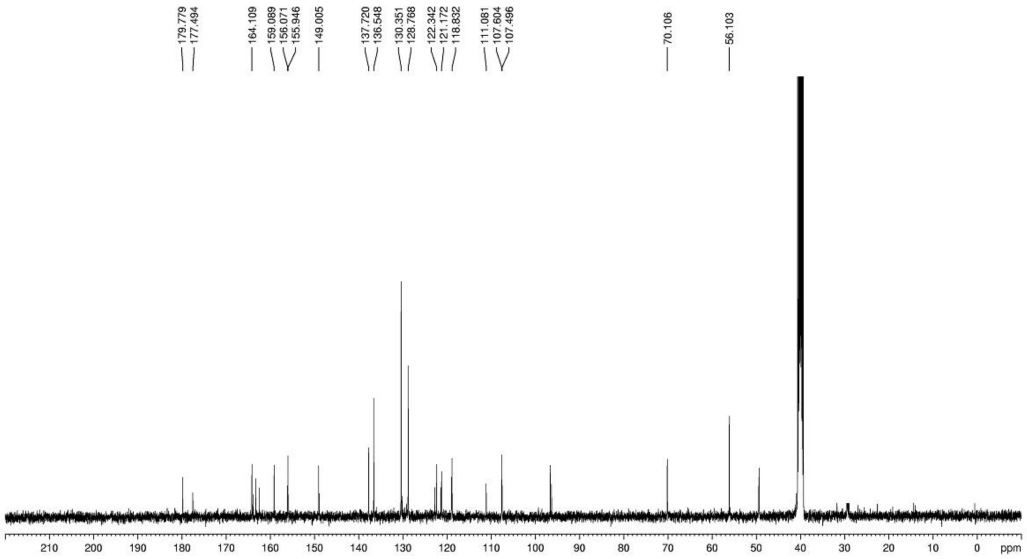

Figure S29. ^{13}C NMR of compound **15a**

Tolerance = 5.0 mDa / DBE: min = -1.5, max = 50.0
Element prediction: Off
Number of isotope peaks used for i-FIT = 3
Monoisotopic Mass, Even Electron Ions
10953 formula(e) evaluated with 114 results within limits (up to 50 closest results for each mass)
Elements Used:
C: 0-50 H: 0-100 N: 0-50 O: 0-100 S: 0-30

Mass	RA	Calc. Mass	mDa	PPM	DBE	Formula	i-FIT	i-FIT Norm	Fit Conf %	C	H	N	O	S
		502.0536	0.8	1.6	25.5	C25 H8 N7 O6	301.6	3.264	3.82	25	8	7	6	
		502.0523	2.1	4.2	20.5	C24 H12 N3 O10	301.7	3.417	3.28	24	12	3	10	
		502.0565	-2.1	-4.2	14.5	C22 H20 N3 O5 S3	303.1	4.825	0.80	22	20	3	5	3
		502.0523	2.1	4.2	31.5	C22 N17	301.0	2.649	7.07	22		17		
		502.0556	-1.2	-2.4	15.5	C21 H16 N3 O10 S	302.5	4.219	1.47	21	16	3	10	1
		502.0556	-1.2	-2.4	26.5	C19 H4 N17 S	302.3	3.988	1.85	19	4	17		1
		502.0534	1.0	2.0	9.5	C18 H24 N5 O2 S5	303.4	5.129	0.59	18	24	5	2	5
		502.0543	0.1	0.2	21.5	C18 H8 N13 O4 S	302.4	4.035	1.77	18	8	13	4	1
		502.0538	0.6	1.2	15.5	C18 H16 N9 O3 S3	303.1	4.758	0.86	18	16	9	3	3
		502.0529	1.5	3.0	3.5	C18 H32 N O S7	303.7	5.371	0.46	18	32	1	1	7

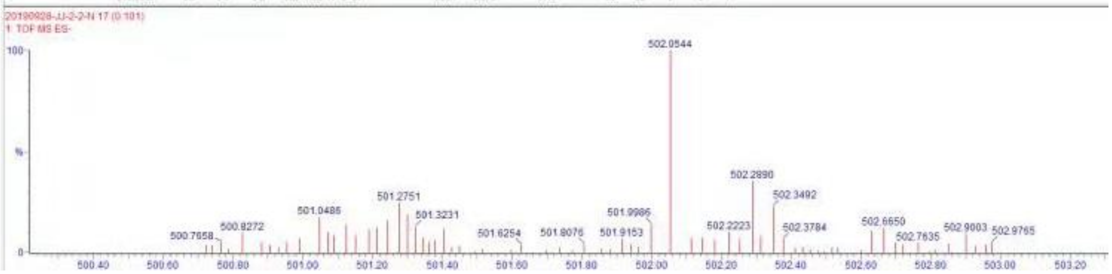

Figure S30. HRMS of compound **15a**

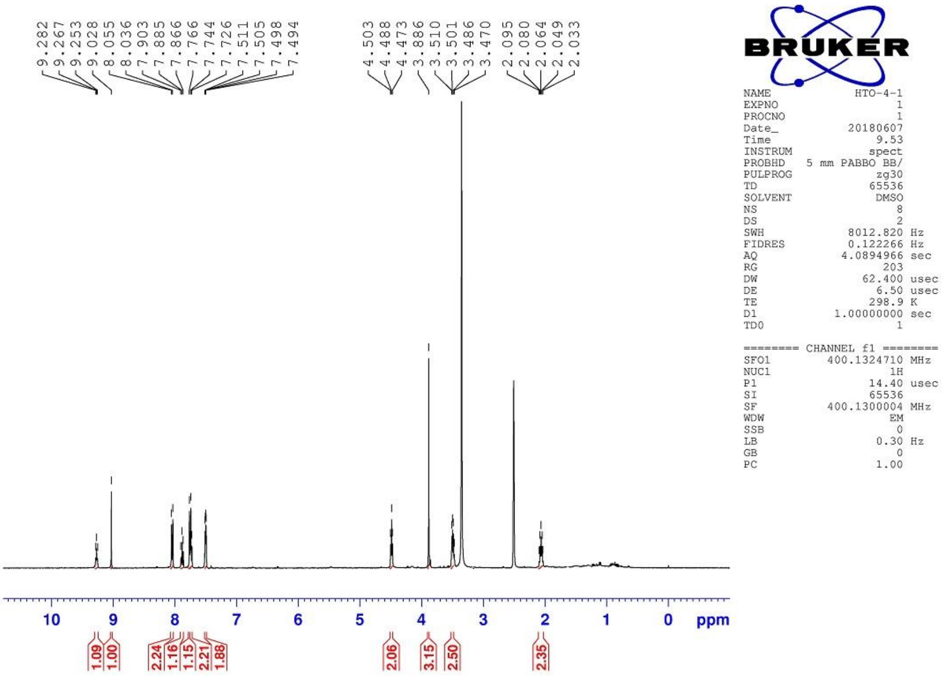

Figure S31. ^{1}H NMR of compound **13b**

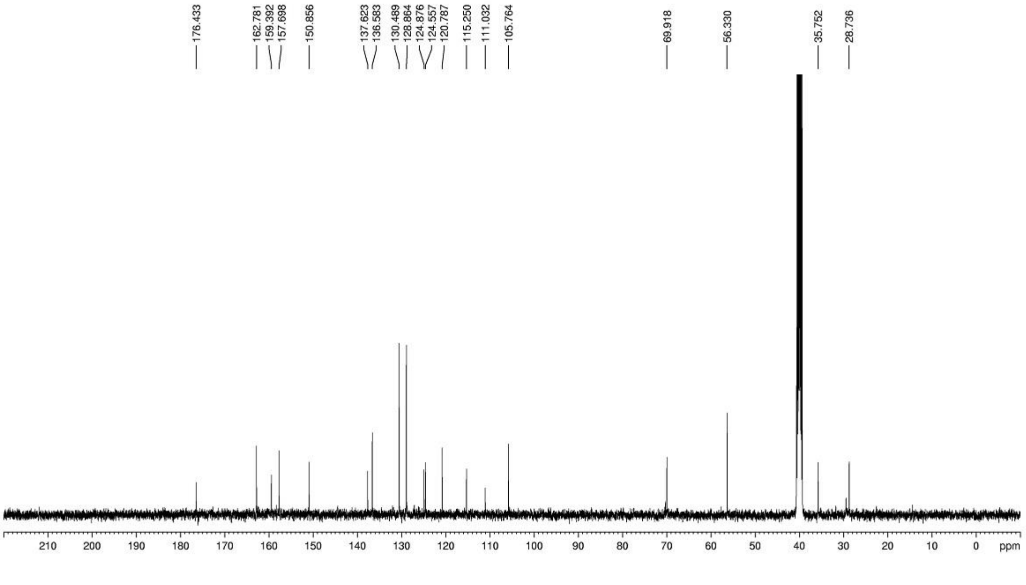

Figure S32. ^{13}C NMR of compound **13b**

Tolerance = 5.0 mDa / DBE: min = -1.5, max = 50.0
Element prediction: Off
Number of isotope peaks used for i-FIT = 3
Monoisotopic Mass, Even Electron Ions
10824 formula(e) evaluated with 108 results within limits (up to 50 closest results for each mass)
Elements Used:
C: 0-50 H: 0-100 N: 0-50 O: 0-100 S: 0-30

Mass	RA	Calc. Mass	mDa	PPM	DBE	Formula	i-FIT	i-FIT Norm	Fit Conf %	C	H	N	O	S
500.0761	100.00	500.0761	0.0	0.0	-0.5	C15 H34 N O5 S6	73.4	4.146	1.58	15	34	1	5	6
		500.0762	-0.1	-0.2	12.5	C14 H14 N9 O12	72.9	3.616	2.69	14	14	9	12	
		500.0762	-0.1	-0.2	23.5	C12 H2 N23 O2	73.0	3.742	2.37	12	2	23	2	
		500.0764	-0.3	-0.6	2.5	C7 H22 N11 O9 S3	73.0	3.693	2.49	7	22	11	9	3
		500.0764	-0.3	-0.6	15.5	C22 H18 N3 O9 S	73.2	3.952	1.92	22	18	3	9	1
		500.0757	0.4	0.8	30.5	C27 H6 N11 O	73.4	4.163	1.56	27	6	11	1	
		500.0757	0.4	0.8	6.5	C14 H22 N5 O11 S2	73.0	3.765	2.32	14	22	5	11	2
		500.0757	0.4	0.8	17.5	C12 H10 N19 O S2	73.1	3.825	2.18	12	10	19	1	2
		500.0766	-0.5	-1.0	5.5	C15 H26 N5 O6 S4	73.3	4.014	1.81	15	26	5	6	4

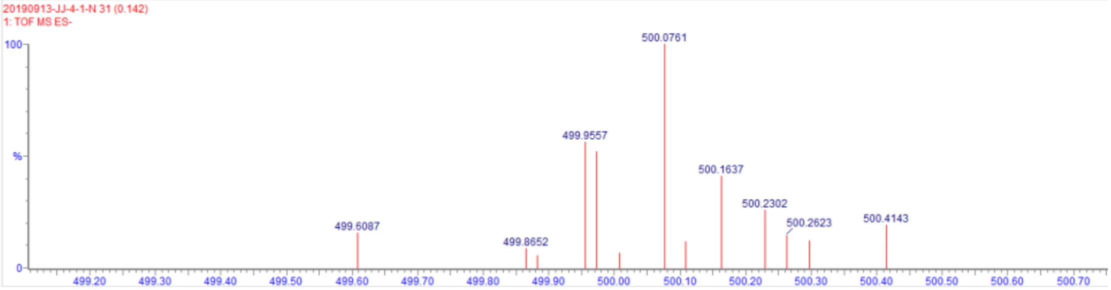

Figure S33. HRMS of compound **13b**

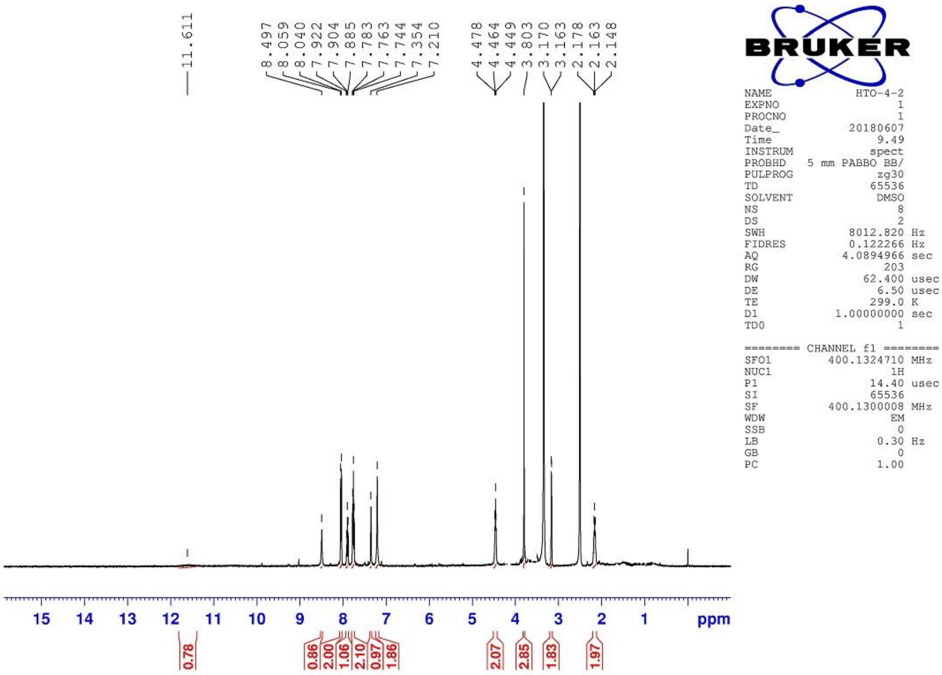

Figure S34. ^{1}H NMR of compound **15b**

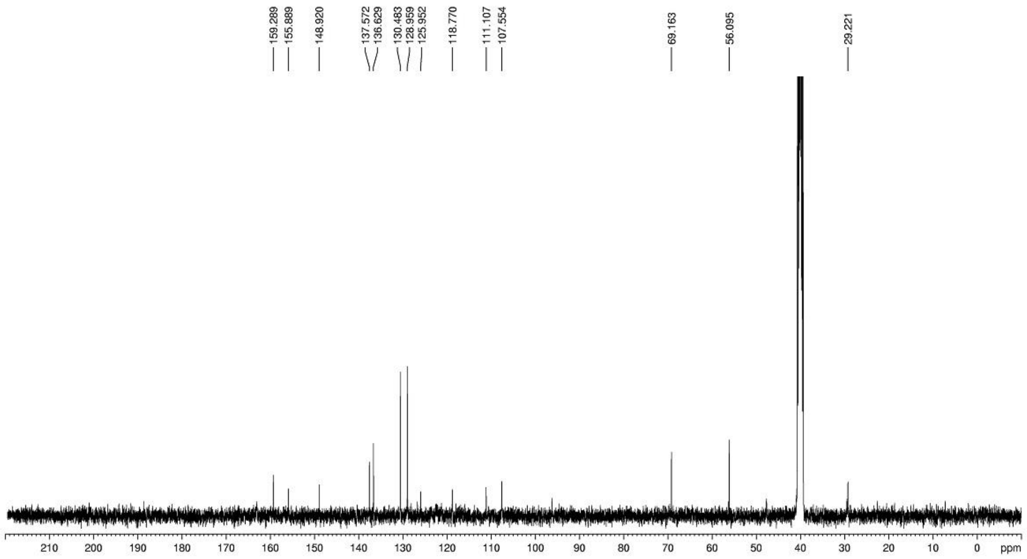

Figure S35. ^{13}C NMR of compound **15b**

Tolerance = 5.0 mDa / DBE: min = -1.5, max = 50.0
Element prediction: Off
Number of isotope peaks used for i-FIT = 3
Monoisotopic Mass, Even Electron Ions
12088 formula(e) evaluated with 116 results within limits (up to 50 closest results for each mass)
Elements Used:
C: 0-50 H: 0-100 N: 0-50 O: 0-100 S: 0-30

Mass	RA	Calc. Mass	mDa	PPM	DBE	Formula	i-FIT	i-FIT Norm	Fit Conf %	C	H	N	O	S
516.0728	100.00	516.0746	-1.8	-3.5	34.5	C32 H6 N9	118.4	4.815	0.81	32	6	9		
		516.0728	0.0	0.0	23.5	C31 H18 N O3 S2	118.0	4.395	1.23	31	18	1	3	2
		516.0733	-0.5	-1.0	29.5	C31 H10 N5 O4	118.0	4.410	1.22	31	10	5	4	
		516.0719	0.9	1.7	24.5	C30 H14 N O8	118.0	4.410	1.22	30	14	1	8	
		516.0740	-1.2	-2.3	25.5	C24 H10 N11 O2 S	118.0	4.419	1.20	24	10	11	2	1
		516.0730	-0.2	-0.4	13.5	C24 H26 N3 S5	118.0	4.414	1.21	24	26	3		5
		516.0735	-0.7	-1.4	19.5	C24 H18 N7 O S3	118.0	4.370	1.26	24	18	7	1	3
		516.0726	0.2	0.4	20.5	C23 H14 N7 O6 S	117.8	4.196	1.51	23	14	7	6	1
		516.0722	0.6	1.2	14.5	C23 H22 N3 O5 S3	117.9	4.321	1.33	23	22	3	5	3
		516.0713	1.5	2.9	15.5	C22 H18 N3 O10 S	118.0	4.388	1.24	22	18	3	10	1

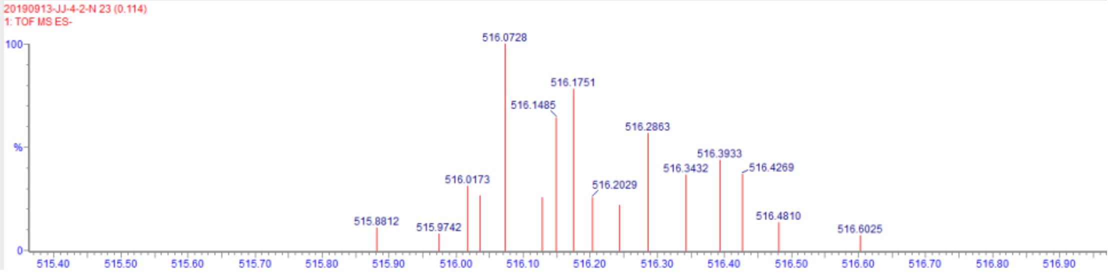

Figure S36. HRMS of compound **15b**

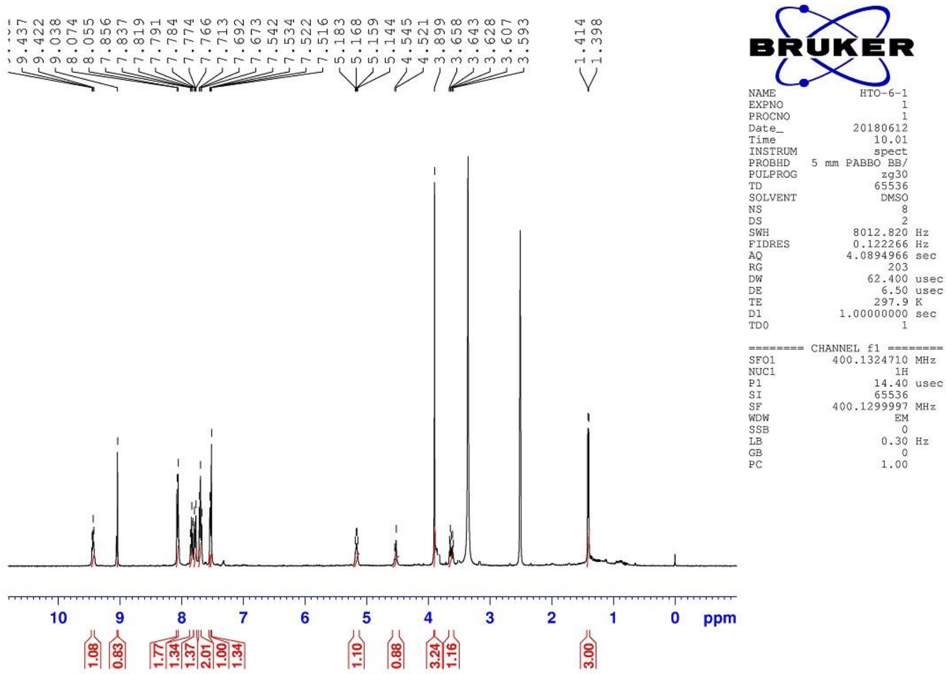

Figure S37. ^{1}H NMR of compound **13c**

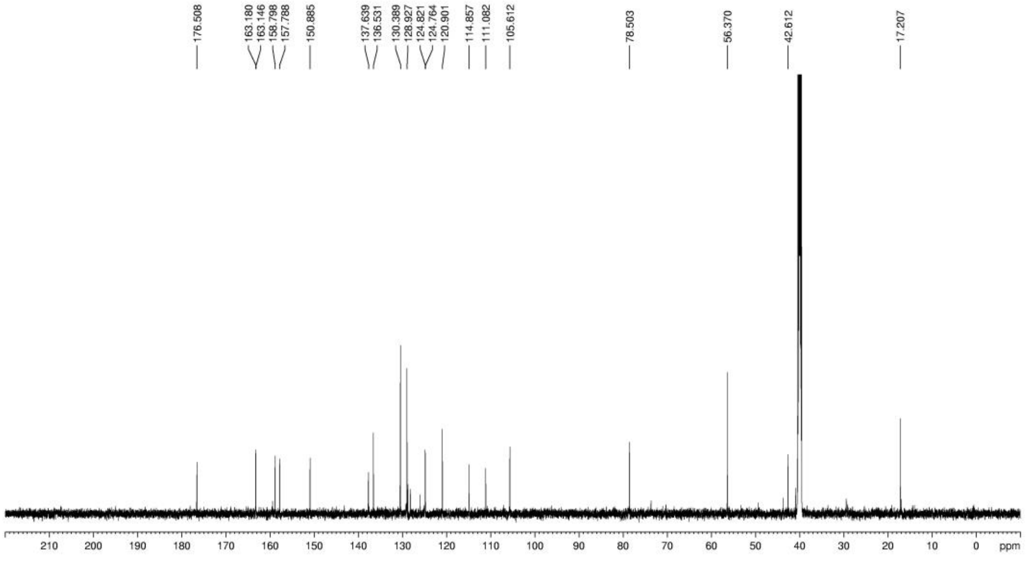

Figure S38. ^{13}C NMR of compound **13c**

Tolerance = 5.0 mDa / DBE: min = -1.5, max = 50.0
Element prediction: Off
Number of isotope peaks used for i-FIT = 3
Monoisotopic Mass, Even Electron Ions
10822 formula(e) evaluated with 107 results within limits (up to 50 closest results for each mass)

Elements Used:
C: 0-50 H: 0-100 N: 0-50 O: 0-100 S: 0-30

Mass	RA	Calc. Mass	mDa	PPM	DBE	Formula	i-FIT	i-FIT Norm	Fit Conf %	C	H	N	O	S
500.0782	100.00	500.0784	-0.2	-0.4	29.5	C31 H10 N5 O3	217.5	4.140	1.59	31	10	5	3	
		500.0779	0.3	0.6	23.5	C31 H18 N O2 S2	217.5	4.162	1.56	31	18	1	2	2
		500.0770	1.2	2.4	24.5	C30 H14 N O7	217.6	4.280	1.38	30	14	1	7	
		500.0804	-2.2	-4.4	19.5	C27 H18 N O7 S	218.0	4.672	0.94	27	18	1	7	1
		500.0786	-0.4	-0.8	19.5	C24 H18 N7 S3	217.4	4.105	1.65	24	18	7		3
		500.0790	-0.8	-1.6	25.5	C24 H10 N11 O S	217.5	4.142	1.59	24	10	11	1	1
		500.0772	1.0	2.0	14.5	C23 H22 N3 O4 S3	217.5	4.171	1.54	23	22	3	4	3
		500.0777	0.5	1.0	20.5	C23 H14 N7 O5 S	217.3	4.016	1.80	23	14	7	5	1
		500.0764	1.8	3.6	15.5	C22 H18 N3 O9 S	217.7	4.367	1.27	22	18	3	9	1
		500.0806	-2.4	-4.8	9.5	C20 H26 N3 O4 S4	218.1	4.802	0.82	20	26	3	4	4

20190913-JJ-6-1-N 14 (0.080)
1: TOF MS ES-

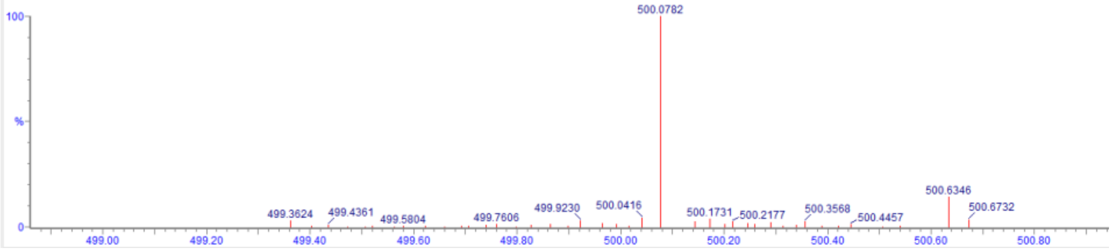

Figure S39. HRMS of compound **13c**

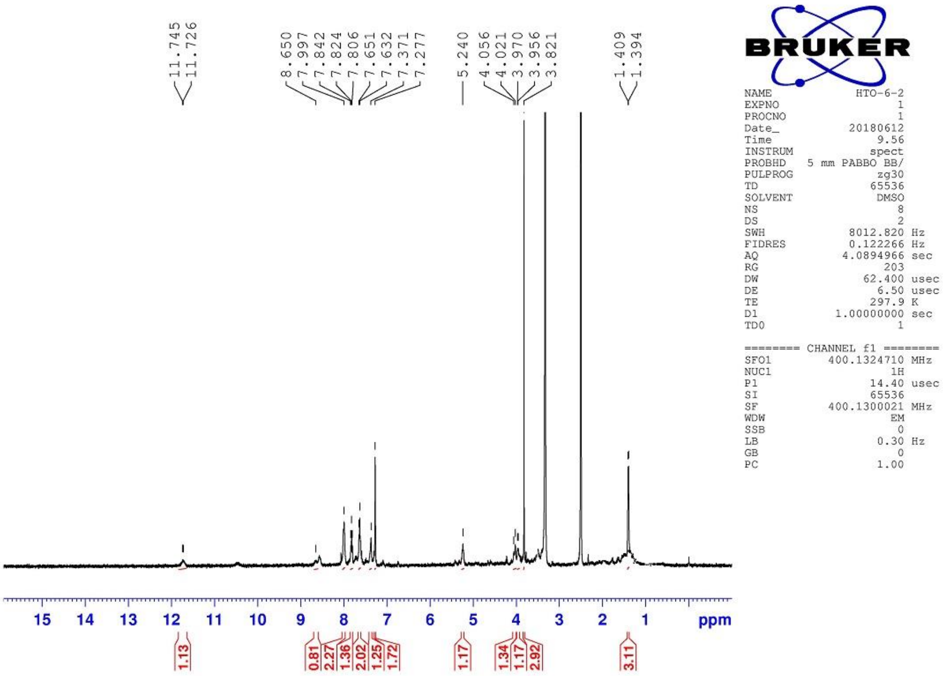

Figure S40. ^{1}H NMR of compound **15c**

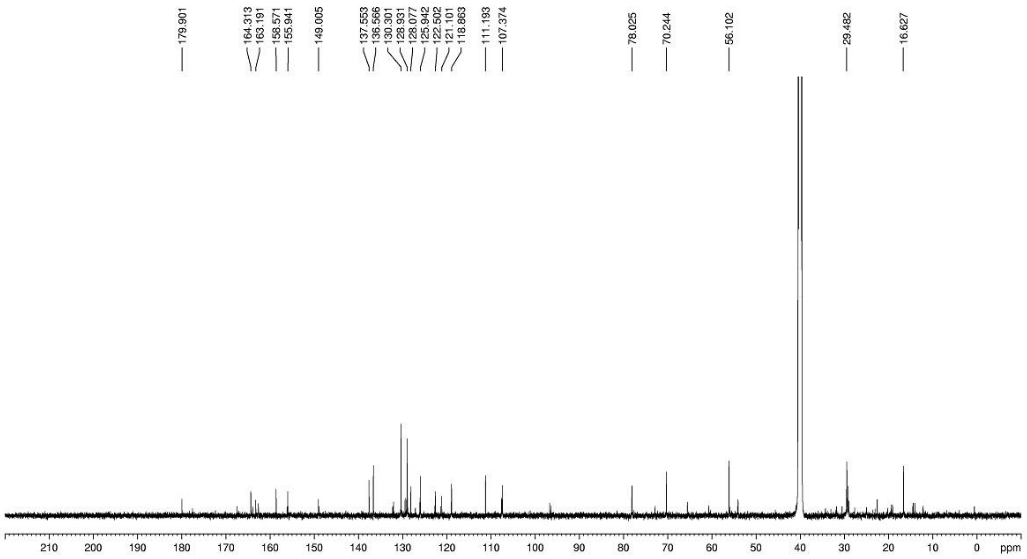

Figure S41. ^{13}C NMR of compound **15c**

Tolerance = 5.0 mDa / DBE: min = -1.5, max = 50.0
Element prediction: Off
Number of isotope peaks used for i-FIT = 3
Monoisotopic Mass, Even Electron Ions
12088 formula(e) evaluated with 120 results within limits (up to 50 closest results for each mass)

Elements Used:
C: 0-50 H: 0-100 N: 0-50 O: 0-100 S: 0-30

Mass	RA	Calc. Mass	mDa	PPM	DBE	Formula	i-FIT	i-FIT Norm	Fit Conf %	C	H	N	O	S
516.0714	100.00	516.0730	-1.6	-3.1	-0.5	C2 H18 N11 O20	158.7	3.083	4.58	2	18	11	20	
		516.0731	-1.7	-3.3	2.5	C10 H22 N5 O17 S	159.4	3.823	2.19	10	22	5	17	1
		516.0698	1.6	3.1	7.5	C13 H18 N5 O17	159.4	3.845	2.14	13	18	5	17	
		516.0705	0.9	1.7	3.5	C6 H18 N11 O15 S	159.0	3.435	3.22	6	18	11	15	1
		516.0711	0.3	0.6	12.5	C14 H14 N9 O13	159.2	3.593	2.75	14	14	9	13	
		516.0706	0.8	1.6	6.5	C14 H22 N5 O12 S2	159.4	3.779	2.28	14	22	5	12	2
		516.0718	-0.4	-0.8	8.5	C7 H14 N15 O11 S	159.0	3.421	3.27	7	14	15	11	1
		516.0702	1.2	2.3	0.5	C14 H30 N O11 S4	159.6	4.066	1.71	14	30	1	11	4
		516.0713	0.1	0.2	2.5	C7 H22 N11 O10 S3	159.2	3.639	2.63	7	22	11	10	3
		516.0713	0.1	0.2	15.5	C22 H18 N3 O10 S	159.4	3.852	2.12	22	18	3	10	1

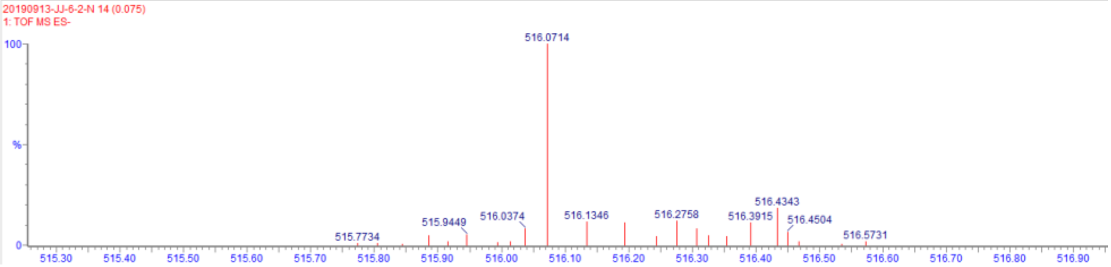

Figure S42. HRMS of compound **15c**

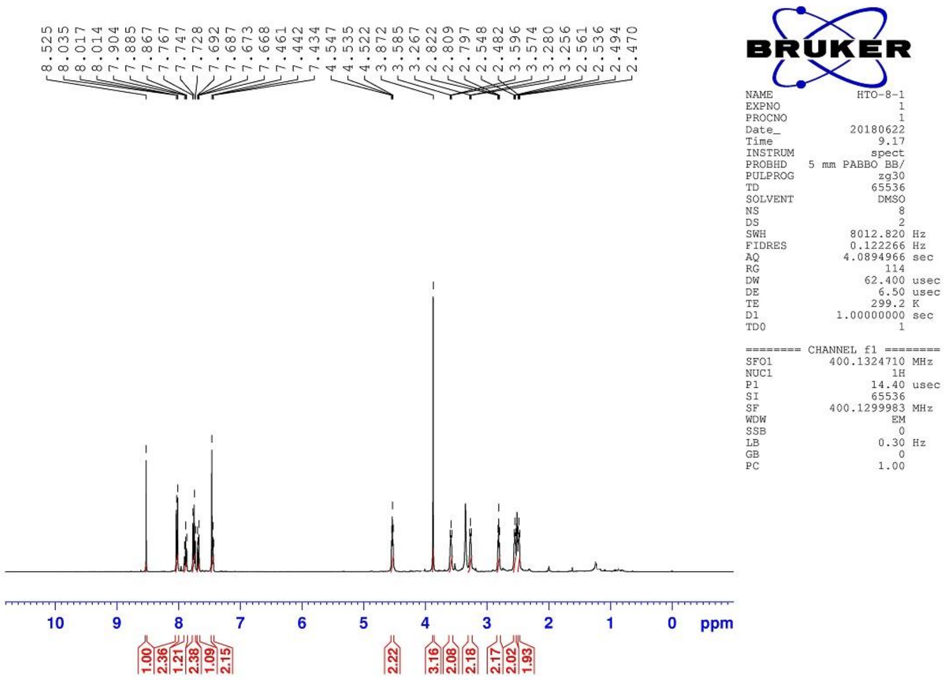

Figure S43. ^{1}H NMR of compound **13d**

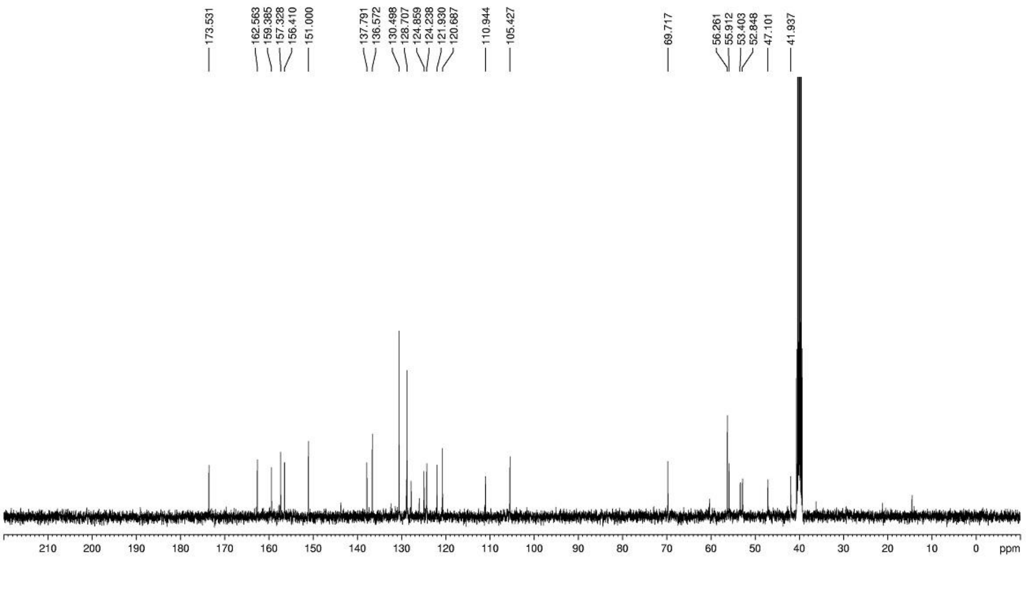

Figure S44. ^{13}C NMR of compound **13d**

Tolerance = 5.0 mDa / DBE: min = -1.5, max = 50.0
Element prediction: Off
Number of isotope peaks used for i-FIT = 3
Monoisotopic Mass, Even Electron Ions
15930 formula(e) evaluated with 136 results within limits (up to 50 closest results for each mass)
Elements Used:
C: 0-50 H: 0-100 N: 0-50 O: 0-100 S: 0-30

Mass	RA	Calc. Mass	mDa	PPM	DBE	Formula	i-FIT	i-FIT Norm	Fit Conf %	C	H	N	O	S
555.1173	100.00	555.1174	-0.1	-0.2	38.5	C45 H15	515.4	0.505	60.36	45	15			
		555.1167	0.6	1.1	29.5	C37 H19 N2 O2 S	518.7	3.827	2.18	37	19	2	2	1
		555.1179	-0.6	-1.1	31.5	C30 H11 N12 O	518.3	3.405	3.32	30	11	12	1	
		555.1174	-0.1	-0.2	25.5	C30 H19 N8 S2	519.6	4.662	0.95	30	19	8		2
		555.1156	1.7	3.1	14.5	C29 H31 O3 S4	519.2	4.315	1.34	29	31		3	4
		555.1161	1.2	2.2	20.5	C29 H23 N4 O4 S2	519.7	4.736	0.88	29	23	4	4	2
		555.1165	0.8	1.4	26.5	C29 H15 N8 O5	518.3	3.367	3.45	29	15	8	5	
		555.1190	-1.7	-3.1	9.5	C26 H35 O3 S5	519.1	4.163	1.56	26	35		3	5
		555.1181	-0.8	-1.4	10.5	C25 H31 O8 S3	519.8	4.883	0.76	25	31		8	3
		555.1186	-1.3	-2.3	16.5	C25 H23 N4 O9 S	519.8	4.837	0.79	25	23	4	9	1

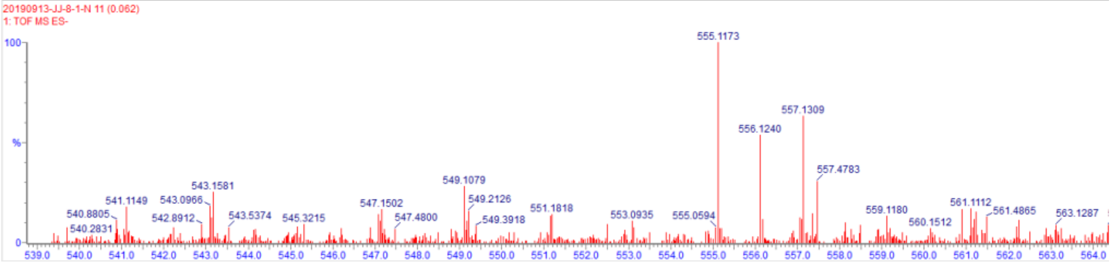

Figure S45. HRMS of compound **13d**

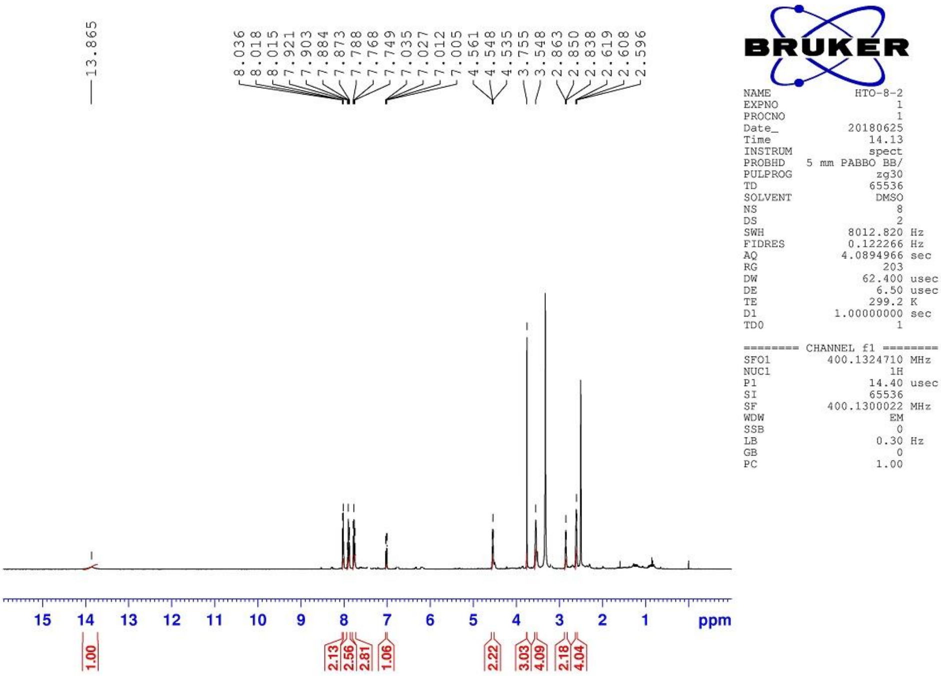

Figure S46. ^{1}H NMR of compound **15d**

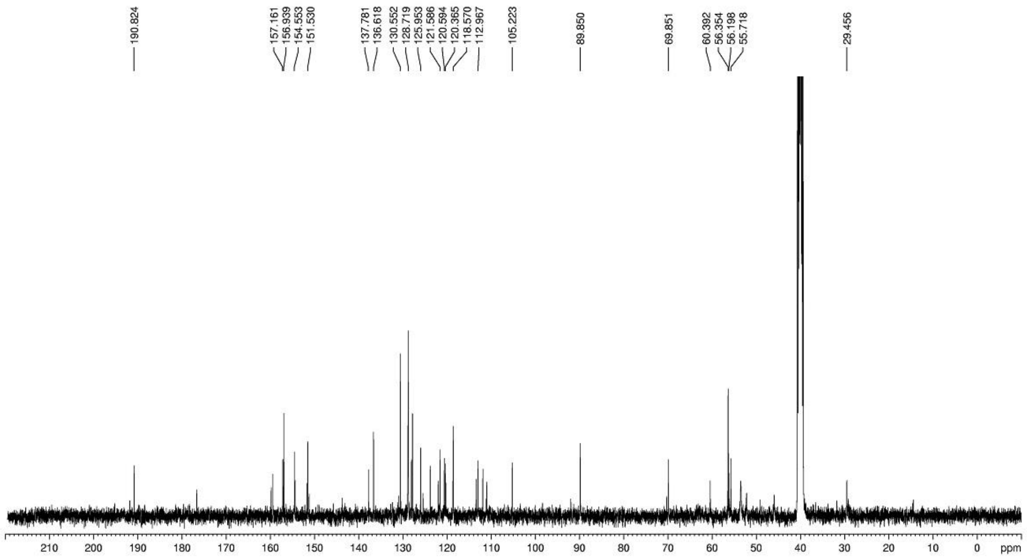

Figure S47. ^{13}C NMR of compound **15d**

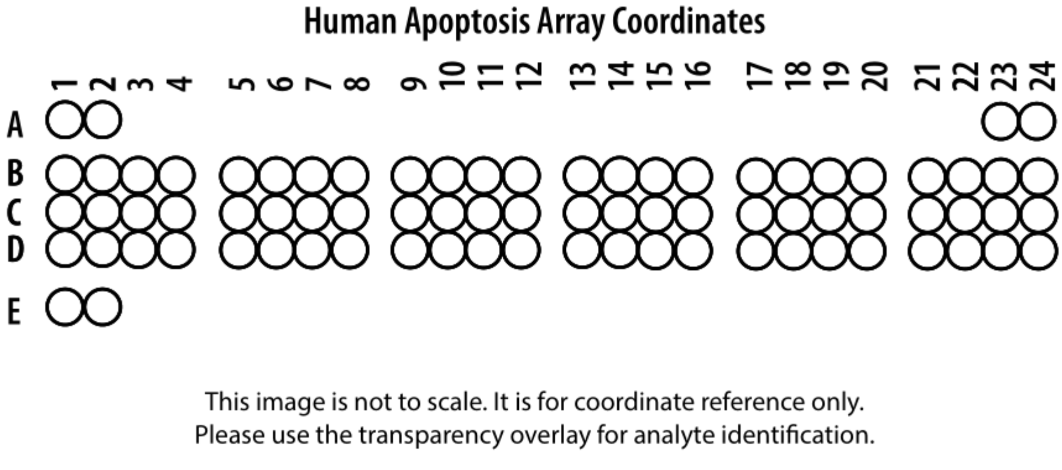

Coordinate	Target/Control	Coodinate	Target/Control
A1, A2	Reference Spots	C13, C14	HO-2/HMOX2
A23, A24	Reference Spots	C15, C16	HSP27
B1, B2	Bad	C17, C18	HSP60
B3, B4	Bax	C19, C20	HSP70
B5, B6	Bcl-2	C21, C22	HTRA2/Omi
B7, B8	Bcl-x	C23, C24	Livin
B9, B10	Pro-Caspase-3	D1, D2	PON2
B11, B12	Cleaved Caspase-3	D3, D4	p21/CIP1/CDKN1A
B13, B14	Catalase	D5, D6	p27/Kip1
B15, B16	cIAP-1	D7, D8	Phospho-p53 (S15)
B17, B18	cIAP-2	D9, D10	Phospho-p53 (S46)
B19, B20	Claspin	D11, D12	Phospho-p53 (S392)
B21, B22	Clusterin	D13, D14	Phospho-Rad17 (S635)
B23, B24	Cytochrome c	D15, D16	SMAC/Diablo
C1, C2	TRAIL R1/DR4	D17, D18	Survivin
C3, C4	TRAIL R2/DR5	D19, D20	TNF RI/TNFRSF1A
C5, C6	FADD	D21, D22	XIAP
C7, C8	Fas/TNFRSF6/CD95	D23, D24	PBS (Negative Control)
C9, C10	HIF-1α	E1, E2	Reference Spots
C11, C12	HO-1/HMOX1/HSP32		

Figure S48. The Human Apoptosis Array coordinates

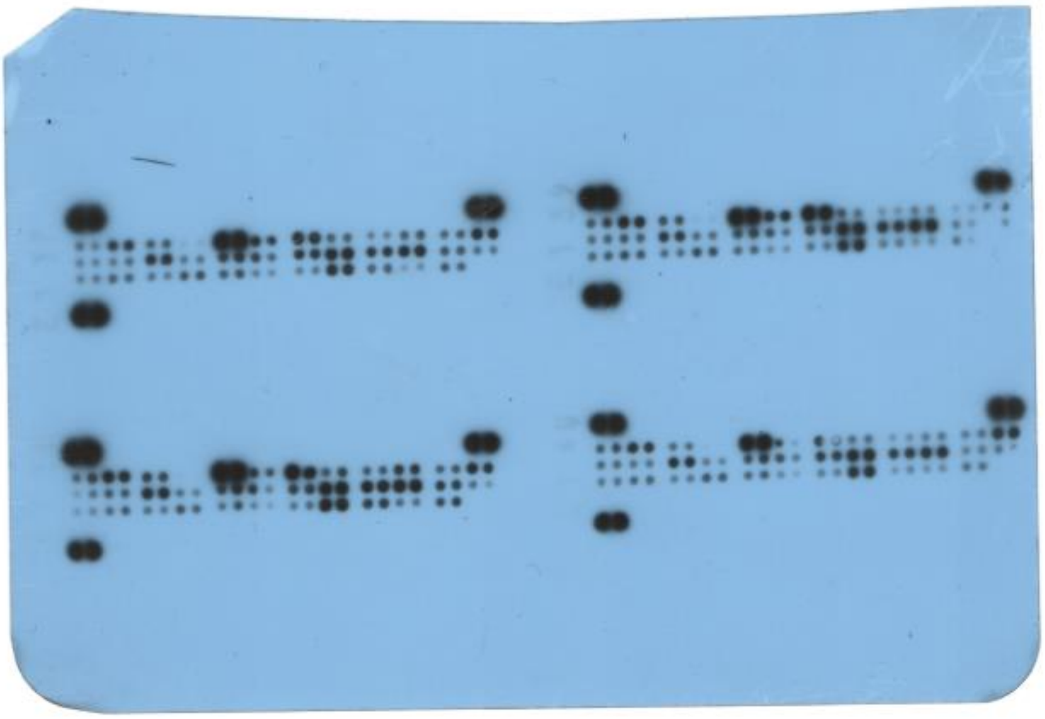

Figure S49. The original image of Human Apoptosis Array kit. The upper left corner was the control group. The bottom left corner was the group incubated with **15a**. The other two groups were other compounds tested simultaneously. The upper right corner was the group incubatied with oridonin derivative. The bottom right corner was the group incubatied with alkaloid derivative.
